# Expanded field of view light-field extended-reality displays with metalens array

**DOI:** 10.1038/s41377-026-02266-w

**Published:** 2026-06-29

**Authors:** Wen-Long Lu, Yang Yi, Zhi-Bin Fan, Shi-Hao Li, Zong Qin, Jian-Wen Dong

**Affiliations:** 1 School of Physics, Sun Yat-sen University, Guangzhou, 510275 China; 2 State Key Laboratory of Optoelectronic Materials and Technologies, Sun Yat-sen University, Guangzhou, 510275 China; 3 School of Electronics and Information Technology, Sun Yat-sen University, 132 East Waihuan Rd, Guangzhou, 510006 China; 4 Guangdong Province Key Laboratory of Display Material and Technology, Sun Yat-sen University, 132 East Waihuan Rd, Guangzhou, 510006 China

**Keywords:** Displays, Metamaterials, Sub-wavelength optics

## Abstract

Extended reality with an immersive visual experience needs to achieve vergence-accommodation-conflict-free (VAC-free) and human-level field of view (FOV) simultaneously. Holography is a dreamlike solution but seriously suffers from both an ultra-narrow diffraction angle and uneliminated coherent laser speckle. Incoherent-based light-field display is an alternative solution, but it still has an inherent issue with off-axis aberration when using a *homogeneous microlens array* for lightweight applications. Here, we propose a *heterogeneous metalens array* (MeLA) that integrates VAC-free operation and aberration correction into a slim monolithic component with expanded FOV. Such a large etendue is due to a unique phase profile in the heterogeneous MeLA, each lenslet of which has an identical hyperbolic term cascading a varying linear-tiling term. Nanoimprint lithography is employed to achieve a centimeter-scale MeLA with a feature aspect ratio of 7:1. In this way, we developed a light-field 3D display prototype with monocular focus cues and a FOV of 50°, approximately four times that of the microlens-array case, and fully exploiting the commercial micro-displays. The proposed MeLA system can even be scaled to human stereoscopic vision, with a maximum value of 80°, without compromising other performance metrics such as depth of field, resolution, and compactness. The characteristics of FOV expansion with monocular 3D display capability are demonstrated through experimental videos generated using a real-time elemental image array algorithm dedicated to the proposed MeLA system. This work opens new opportunities for extended reality through VAC-free 3D display and a wide FOV, poised to transform consumer electronics, precision nanofabrication, and healthcare.

## Abstract (web summary)

XR 3D display prototype achieves VAC-free experience via unique heterogeneous metalens array, offering extended FOV for next-gen AI glasses and outdoor navigation in smart wearables.

## Graphical abstract

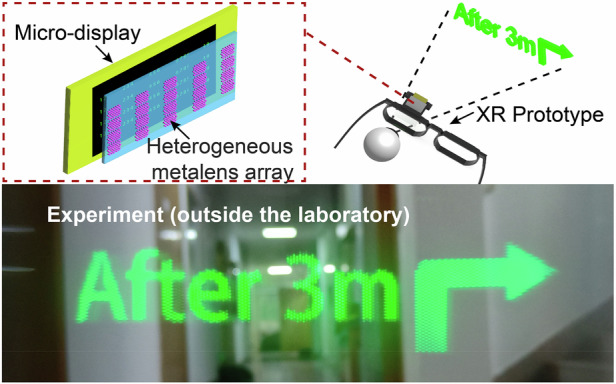

## Introduction

Extended reality (XR) near-eye displays (NEDs) can transform consumer electronics, industrial manufacturing, healthcare, etc^1–5^. by mixing virtual information and the physical world. Compared with commercialized augmented reality, XR NEDs feature two indispensable improvements. First, XR NEDs should create realistic monocular 3D scenes with mitigated vergence-accommodation conflict (VAC). VAC-free 3D display technologies such as digital holography^6–10^ and light-field displays^11–26^ can be considered. Next, the field of view (FOV) of virtual information should match the physical world for seamless mixing. The FOV of early augmented reality glasses only ranged from 20 to 30°. Nowadays, augmented reality products available on the market can offer a FOV of approximately 40°, whereas further expanding the FOV is limited by the nature of light, such as aberrations and diffraction. Some studies^27,28^ have achieved FOVs greater than 60° using a metalens eyepiece. However, the VAC has rarely been addressed in the works on FOV expansion. Either 2D images with a wide FOV or a VAC-free 3D display with a limited FOV. The former one may add a quasi-3D experience through binocular parallax, which, however, is well known to cause the VAC. Regarding the requirement for XR displays beyond current augmented reality glasses, achieving VAC-free 3D display and a wide FOV simultaneously in NEDs is eagerly sought in a monolithic, compact device. To our knowledge, such a NED without the FOV-VAC dilemma has been little reported in academic research, let alone in commercial products.

Digital holography and integral imaging light-field displays are promising VAC-free 3D display technologies, but both suffer from a narrow FOV. The FOV of digital holography based on spatial light modulators is limited by the diffraction angle, intrinsically requiring a smaller pixel size that poses manufacturing challenges. In contrast, metasurface holography offers a path to wider FOVs by utilizing subwavelength nanostructures^29,30^. On the other hand, the FOV of light-field NEDs is limited by the microlens array’s aberrations. Considering the light-field NED’s advantages, such as compact volume, coherent source-free, and moderate computation, addressing the aberration-induced FOV issue is attractive for practical XR NEDs. Multiple strategies have been reported for the narrow FOV issue in light-field displays, including multiple projection units^11–13^, curved microlens array^14–16^, gaze-tracking assisted image rendering^17,18^, and light control components^19–23^. However, these solutions may increase system volume or only apply to glass-free scenarios rather than NEDs. Some studies combined a light-field 3D engine and a freeform prism eyepiece to provide see-through 3D images with an approximately 30-degree FOV^24,25^. Further expanding the FOV of the NED is impeded by the eyepiece’s etendue. Since human stereoscopic vision supports 3D depth cues extending to at most 80°^31^, making lightweight light-field NEDs with stereopsis-matching FOV is an enduring challenge.

Recently, metalenses^26–28,32–53^ have exhibited significant potential in imaging and display due to their lightweight volume, flexible light manipulation, and multi-functionality. For example, researchers proposed metalenses for NEDs^27,28,35–37^ and wide FOV imaging^38–40^. Additionally, *homogeneous* metalens arrays (MeLAs) have been utilized for light-field imaging^41–43^, while *heterogeneous* MeLAs enable wide-angle cameras with expanded FOV through their tailored optical phase distributions^44,45^. Especially, our previous work^26^ proposed a *homogeneous* MeLA-based light-field NED, pioneering the application of metalenses in VAC-free 3D NEDs. Despite the potential of metalenses, their limited fabrication size remains a key challenge. Wide FOV means a large etendue, necessitating larger metalenses (or arrays). For large-scale metalenses, nanoimprint lithography^46–48^ provides a low-cost approach, with wafer-scale MeLAs already demonstrated^49–51^. More innovatively, water-soluble molds have enabled the fabrication of nanoimprinted metalenses with a high aspect ratio (6:1)^52^. These breakthroughs support next-generation NEDs; however, nanoimprinting for high-aspect-ratio MeLAs with high-refractive-index (high-n) materials remains challenging.

This study proposes a heterogeneous MeLA. Based on this array, we have developed a light-field NED with an expanded FOV. In contrast to the conventional microlens array with identical lenslets, the lenslet’s phase profile of the proposed MeLA spatially varies to precisely correct aberrations across the overall FOV. The general concept of the aberration correction is a complex function of space-variant prisms and lenses. Corresponding to the heterogeneous lenslets, a new algorithm for generating elemental image arrays has been developed to upgrade the conventional practice that performs space-invariant viewpoint projection. Moreover, the heterogeneous MeLA is fabricated through nanoimprint with a high-refractive-index imprint material, featuring a maximum aspect ratio of 7:1 in its nanostructures. Nanoimprint lithography fabrication enables a sufficient dimension of the MeLA to match a commercial Si-OLED micro-display. As a result, the proposed meta-based light-field NED experimentally achieves a 50-degree FOV, approximately four times that of traditional light-field NEDs. An 86-degree FOV is also simulated for demonstration of scalability, which is sufficient to encompass the human stereoscopic vision range (80°).

## Results

### Optical architecture based on geometric optical elements

**Fig. 1:**
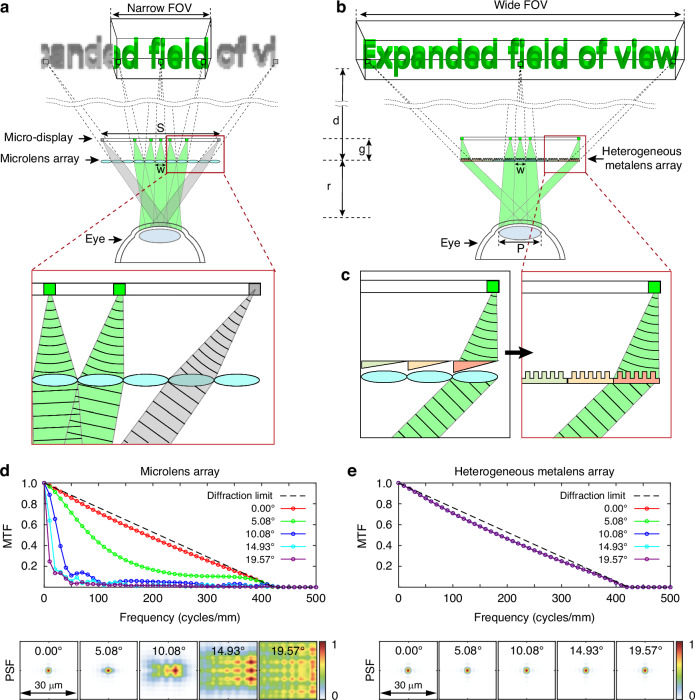
. **Schematic illustration of the principle of expanded FOV**. **a** Conventional light-field NED based on a homogeneous microlens array. The central green region illustrates that light from the pixels enters the eye in a state of paraxial imaging, maintaining acceptable image quality. This corresponds to the narrow *FOV*_0_, indicated by the green font above. The surrounding gray regions represent that light from the pixels enters the eye in a state of off-axis imaging, resulting in severe aberrations, which corresponds to the gray-marked *FOV* range. **b** The proposed FOV-expanded light-field NED. The lenslets at different positions in the MeLA exhibit heterogeneous designs, functionally equivalent to superimposing prisms to varying angles on the lens. The entire screen meets the near-diffraction-limited imaging condition, and the corresponding high-quality FOV range at the top has been significantly increased. **c** Schematic diagram of the combined function of an identical lens array and a heterogeneous prism array, which can deflect the light from pixels by a certain angle. **d** MTFs and PSFs degradation with five field angles in a conventional microlens array. **e** Constant MTFs and PSFs across five angular fields in the heterogeneous MeLA, demonstrating near-diffraction-limited performance in an extended FOV

Conventional light-field NED consists of a micro-display and a microlens array, as illustrated in Fig. 1a, where the lenslets are identical. The elemental image array on the micro-display is projected into the human eye through the microlens array. Elaborate parallaxes recorded in the elemental image array enable the perception of 3D virtual images. In this conventional configuration, the overall FOV of the virtual image is limited by the off-axis aberration of the lenslets. A qualified microlens array can typically maintain acceptable imaging (green front) within its individual *FOV*_0_, as given by Eq. (1).

1 $$FO{V}_{0}=2\cdot \arctan \left(\frac{w}{2g}\right)$$

where *w* is the lenslet pitch, and *g* is the gap between the microlens array and the micro-display. An elemental image placed outside *FOV*_0_ produces severe aberration (gray region). Unfortunately, *FOV*_0_ is usually no greater than 20°, as a simple spherical lens profile is employed. For example, a typical microlens array has *w* = 400 μm and *g* = 1.8 mm, i.e., a numerical aperture (NA) of 0.11, so the overall *FOV*_0_ is limited to only 13°. To quantitatively evaluate imaging performance, we calculated modulation transfer functions (MTFs) and point spread functions (PSFs) of the simple spherical lens at five angular fields. As shown in Fig. 1d, both the MTFs and PSFs significantly degrade across the five angular fields, failing to meet the wide FOV requirement. In particular, the pixel size of current micro-displays is around five to ten microns, only slightly larger than the diffraction limit of the lenslets (0.61λ/NA ~ 3 μm). To fully leverage the high pixel density of these micro-displays, the optical resolution of the microlens array must be fine enough to resolve individual pixels. Therefore, working in a near-diffraction-limited status is essential for the microlens array. Regarding FOV expansion, a larger *FOV*_0_ (namely, a larger NA) is challenging for traditional microlens arrays. Also, a larger NA reduces the depth of field, which is adverse to the depth range the light-field NED can render.

Another limitation of the overall FOV is the dimension of the microlens array. The microlens array dimension positively correlates with the FOV and eyebox size (i.e., the etendue from their product). The larger the FOV, the larger the microlens array size required. Thus, the microlens array size should be a couple of centimeters under a standard eyebox of one to two centimeters and a FOV measuring tens of degrees. Such a dimension may hinder electron-beam lithography-based metalenses; therefore, large-area fabrication processing, such as nanoimprint lithography, is necessary.

One straightforward approach to FOV expansion is to use an off-axis aberration-corrected lens array; however, its requirement for multiple lens elements makes it impractical for lightweight NEDs. We observe that the lenslets generally correspond to segmented FOVs; e.g., the central lenslet is mainly responsible for the central FOV segment. Based on this observation, we propose a concept of spatially heterogeneous MeLA, where each lenslet has a distinct phase profile to guarantee near-diffraction-limited imaging within its corresponding FOV segment. At oblique fields, a spherical phase profile cannot eliminate off-axis aberrations; thus, we further propose that the distinct phase profile is comprised of a spherical plus a linear phase profile to function as a lens cascading a prism, as shown in Fig. 1c. The linear phase profile deflects a beam so that the beam penetrates the spherical phase profile at a paraxial state. In this way, the off-axis imaging task is split into two parts. The optical power is dominantly carried by the paraxial lens; light deflection is performed by the prism with minimized aberrations. MTFs and PSFs are evaluated for the heterogeneous MeLA at the five angular fields by adding a prism to a spherical lens, as shown in Fig. 1e. As a result, compared to the conventional microlens array, the proposed heterogeneous MeLA achieves near-diffraction-limited performance over a significantly larger FOV.

The specific design needs to determine the deflection angle *α*_*i*_ of each microprism. To this end, ray tracing is performed from each metalens center to the pupil center. It is crucial to note that this deflection angle *α*_i_ is not identical to the system’s FOV. The detailed mathematical relationship between the set of deflection angles *α*_*i*_ and the final system FOV is discussed in Section 1 of the Supplementary Information. The overall FOV can be calculated by Eq. (2),

2 $$FOV=2\cdot \arctan \left(\frac{S-w+P}{2r}\right)$$

where the micro-display size *S* is 13.2 mm, the pupil size *P* is 4 mm, the lenslet pitch *w* is 400 μm, and the eye relief *r* is 18 mm. As a result, the FOV is approximately 50° according to Eq. (2). Meanwhile, the FOV of the conventional light-field NED, limited by each lenslet, is only 13°. Subsequent MeLA design and experiments will aim to achieve a 50-degree FOV, as determined by the micro-display in hand. Here, we should emphasize that when considering human stereoscopic vision spans 80° maximally, a much larger screen size of a micro-display needs to be applied. We will provide a feasible design for an 86-degree FOV in Section 2 of the Supplementary Information to demonstrate that our heterogeneous MeLA solution is scalable to a human-level experience.

### Heterogeneous metalens array

**Fig. 2:**
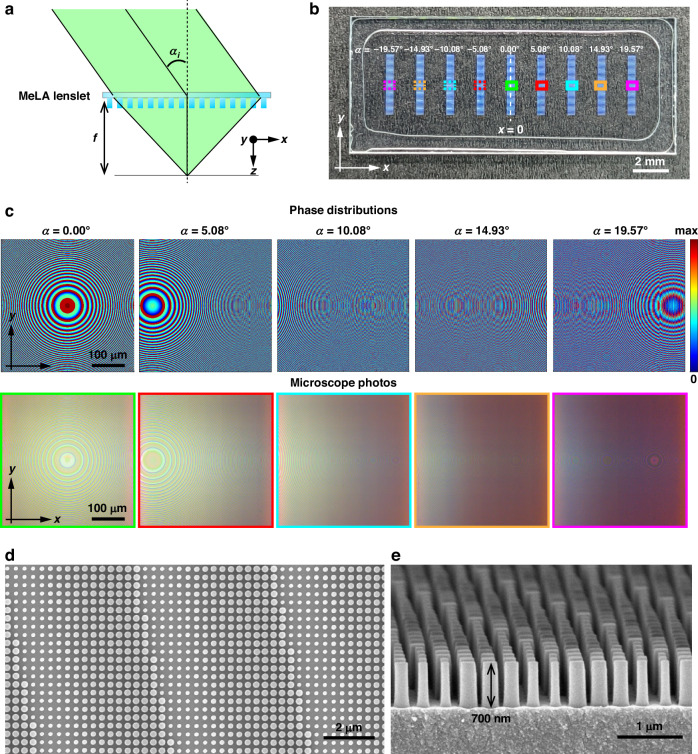
. **Design principle and morphology of nanoimprint MeLA**. **a** Schematic illustrating the principle of the MeLA lenslet, which focuses obliquely incident light to a designated focal point. **b** Photographs of a nanoimprinted MeLA sample, scale bar, 2 mm. The entire array is designed to be symmetric about the central axis (x = 0). Dashed and solid boxes in matching colors highlight pairs of metalenses with equal but opposite deflection angles. **c** Comparison of designed phase distributions (top row) and their corresponding fabricated structures imaged by an optical microscope (bottom row) for five representative lenslets. Each profile is unique, corresponding to a specific target deflection angle. The microscope photos show zoomed-in regions corresponding to the solid-box areas marked in (**b**). The consistency between the top and bottom rows visually demonstrates the successful transfer of the designed phase patterns into physical structures. **d** Top-view and **e** cross-sectional SEM images of a portion of the MeLA. The top-view image shows the highly ordered, periodic arrangement of nanopillars with varying diameters. The cross-sectional view reveals the vertical, cylindrical morphology of individual nanopillars, confirming their uniform height

Although the composite phase profile results in a bulky multi-element group in geometric optics, a monolithic metalens can engineer its nanostructures to provide a complex phase response. Hence, we propose the heterogeneous MeLA, as shown in Fig. 2a, which is functionally equivalent to the heterogeneous lens array described above without increasing fabrication difficulty. The core component is the MeLA, with the engineered phase response functioning as a lens cascaded with a prism; the prism has a spatially variant deflection angle. We extend the horizontal FOV and leave the vertical direction for comparison. The phase distribution of the i-th lenslet can be written as Eq. (3),

3 $$\begin{array}{l}{\varphi }_{\mathrm{meta},i}=-\frac{2\pi }{\lambda }\left(\sqrt{{x}^{2}+{y}^{2}+{f}^{2}}-f\right)-\frac{2\pi }{\lambda }x\cdot \,\sin ({\alpha }_{i})+c\\ {\mathrm{with}}\,{\alpha }_{i}=\arctan \left(\frac{{x}_{ic}}{r}\right)\end{array}$$

where *x* and *y* denote the pupil coordinates on a unit of the MeLA; the focal length *f* is 1.8 mm; *λ* is the working wavelength of 525 nm; *c* is a constant, and *x*_*ic*_ denotes the horizontal coordinates of the *i*-th lenslet center. The deflection angle *α*_*i*_ denotes the position *x*_*ic*_ of the *i*-th unit relative to the eyebox center. The *i*-th lenslet can converge the plane wave incident at angle *α*_*i*_ to the center directly below the lenslet, as shown in Fig. 2a.

For the nanopillar design, the angular dependence of the phase response should be considered rather than normal light. Structural parameters are screened out according to the conditions of weaker coupling between nanopillars and no higher-order diffraction. The nanopillars are made of high-n (*n* = 1.91@525 nm) imprint material to minimize coupling, while the grating period is reduced to suppress higher-order diffraction, thereby maximizing transmittance. In addition, a high-aspect-ratio nanopillar can achieve broader phase coverage but increases fabrication difficulty. After careful consideration, this study limits the maximum aspect ratio of the nanopillars to 7:1. Finally, the nanopillar grating’s period is 330 nm, the nanopillar diameter ranges from 100 to 230 nm, and the height is 700 nm. More details are given in Section 3 of the Supplementary Information. The designed MeLA spans 13.2 by 3.2 mm, with a lenslet pitch w = 400 μm, comprising 33 columns and eight rows. To simplify fabrication, we selectively retained nine complete columns, as illustrated in Fig. 2b. This discretized MeLA achieves FOV expansion equivalently to the fully populated configuration. To cover a wide range of horizontal FOV, columns of lenslets were engineered with a discrete set of design deflection angles (*α*_i_ = 0.00°, ±5.08°, ±10.08°, ±14.93°, and ±19.57°). Each set of lenslets with a specific *α*_i_ is responsible for forming a portion of the total FOV. Figure 2c shows the phase distributions of the five lenslets (top) and their corresponding microscope photos (bottom). These microscope photos are zoomed in on regions corresponding to those shown in Fig. 2b. As shown in Fig. 2c, the metalenses engineered for different horizontal FOVs exhibit distinct phase distributions. The microscope photos show strong correspondence between the designed phase patterns and the physical patterns formed by the nanopillar arrangements, indicating a high-fidelity fabrication process. The specific fabrication process is described in Section 4–5 of Supplementary Information. Regarding the bubble-like defect in high-n, high-aspect-ratio nanoimprint, we integrated vibration during stamp coating, combining gravity and vibration to achieve complete air-free filling. Subsequent standard fabrication steps (spin coating, exposure, demolding) yielded intact samples with defect-free nanostructures, as evidenced by SEM analysis. Figure 2d, e present the top-view and cross-sectional SEM images of a selected sample area, respectively. These images reveal that nanopillars of varying diameters exhibit excellent morphology and are arranged in a well-ordered, periodic array, as designed. Measurements confirm that the diameters and heights of the nanopillars are in excellent agreement with the design layout, demonstrating the high fidelity of our fabrication process at the microscopic level.

**Fig. 3:**
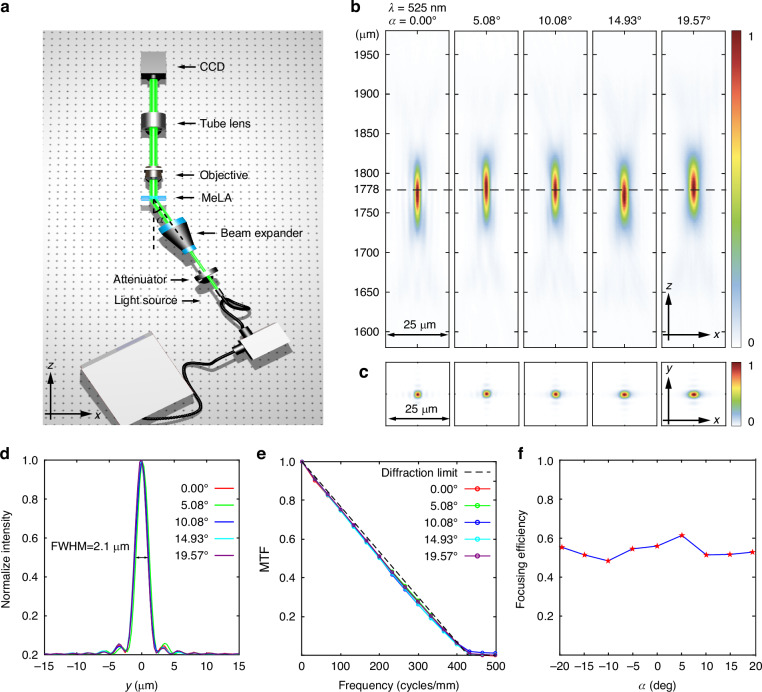
. **Optical performance characterization of the fabricated MeLA**. **a** Schematic of the optical setup used for characterization. A collimated 525 nm laser beam is directed at specific incident angles (*α*_*i*_) onto the corresponding MeLA lenslets. **b** Normalized measured intensity distributions in the *x*-*z* plane for five types of MeLA lenslets. The plot shows that obliquely incident light is effectively deflected and focused onto the central axis, with an average focal length of 1778 μm. **c** Normalized measured intensity distributions at the focal plane corresponding to each lenslet in (**b**). **d** Cross-sectional intensity profiles of the focal spots along the y-direction. The profiles for all five lenslets are nearly identical and tightly overlap with each other, with a measured full width at half maximum (FWHM) of 2.1 µm. **e** MTFs derived from the curve profiles in (**d**). The performance of all tested lenslets closely approaches the theoretical diffraction limit (black dashed curve). The cut-off frequency is measured at 330 cycles/mm (MTF = 0.2). **f** The measured focusing efficiencies of nine lenslets, showing relative consistency across the array with a mean value of 54%

The fabricated MeLA was characterized using 525 nm illumination with a detailed configuration shown in Fig. 3a. After collimation and expansion, the light is incident at different angles α_*i*_ onto the corresponding lenslets. The light intensity distributions of the five lenslets in the *xz*-plane are shown in Fig. 3b. Obliquely incident light is focused on the central axis by the lenslets, validating the composite phase response function of a lens and a prism. All lenslets focus light almost on the same focal plane, with an average focal length of 1778 μm and an average depth of focus of 49 μm, consistent with the design. Figure 3c shows the normalized intensity distributions on the focal plane corresponding to Fig. 3b. Figure 3d shows the intensity profile of the focal spot along the *y*-direction, revealing that the PSFs of MeLA units for different deflection angles overlap significantly. The full width at half maximum measures only 2.1 μm, much smaller than the micro-display’s pixel size (~8 μm), demonstrating the high enough resolution of the MeLA sample. Figure 3e shows the MTFs corresponding to the cross-sectional intensity in Fig. 3d, where the black dashed curve indicates the diffraction limit. The plot reveals that all lenslets, designed for different deflection angles, exhibit near-diffraction-limited performance. The cut-off frequency is 330 cycles/mm, at which the MTF drops to 0.2. The focusing efficiencies of individual lenslets under incident light at corresponding angles are shown in Fig. 3f. The units exhibit relatively consistent focusing efficiencies, with an average of 54%. This level of uniformity is advantageous for maintaining consistent brightness across the light-field display. For more details, see Section 6 of Supplementary Information.

### Verification of FOV expansion

**Fig. 4:**
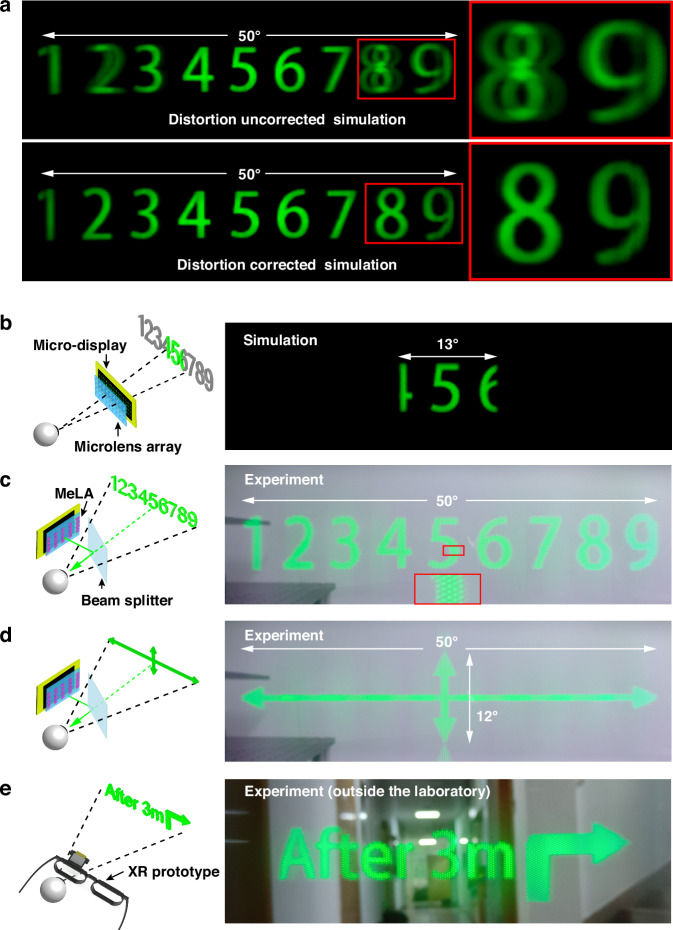
. **Demonstration of FOV expansion display**. **a** Comparison of rendering algorithms on the meta-based light-field NED. Top: Distortion from the conventional elemental image array rendering algorithm. Bottom: The proposed elemental image array rendering correction algorithm achieves uniform imaging across the whole FOV (50°). **b** Optical path diagram and simulation of conventional light-field NED show that the FOV is just 13° and only parts of numbers 4–6 can be displayed. **c** Optical path diagram and experiment of the FOV-expanded light-field NED, realizing an expanded FOV of 50° that can display all the numbers 1–9. This is consistent with the simulation result in (**a**). **d** Horizontal vs. vertical FOV comparison for the FOV-expansion. The vertical FOV, which was not targeted for expansion, remains at 12°, similar to that of conventional architectures. The displayed image of continuous arrows demonstrates that the carefully engineered discrete MeLA is capable of producing a seamless visual field. **e** Schematic of the wearable XR glasses prototype and a demonstration of its XR levitation navigation effect, outside the laboratory, which displays virtual navigation information in front of the human eye at a distance of distinct vision with finite distance of approximately 25 cm

The generation process of the elemental image array can be considered an imaging process, while spatially varying prismatic phases in the MeLA introduce distortions in the imaging. The conventional elemental image array generation algorithm becomes invalid because the geometric projection relationship is not established for each lenslet. We therefore established a dedicated voxel-to-pixel mapping for MeLA. We proposed a specialized algorithm to generate elemental image arrays for meta-based light-field NED, addressing display distortions through preprocessing (see Section 7 in the Supplementary Information for more details). Figure 4a compares elemental image arrays generated by different rendering algorithms on the proposed light-field NED. As shown in Fig. 4a (top), when the conventional elemental image array generation algorithm is applied, the digits “4 5 6” in the central FOV are reconstructed normally, whereas the digits “1”, “2”, “8”, and “9” near the edge FOV exhibit severe splitting and distortion increasing with the FOV. As shown in Fig. 4a (bottom), using the proposed correction algorithm, both the edge and central digits are correctly reconstructed. Since fast rendering of conventional light-field displays is guaranteed by spatially uniform geometric projections, adopting the MeLA should not increase computational complexity. To this end, we introduce a lookup table that maps voxels to pixels to circumvent space-variant ray tracing in the MeLA^26,54^. Our algorithm achieves a rendering speed of 140 FPS on an ordinary laptop (AMD Ryzen 7-7840HS) by leveraging the lookup table.

To demonstrate FOV expansion, we compare the FOV of the conventional light-field NED with the proposed one. Figure 4b–e shows optical path diagrams (left) and corresponding simulated/experimental results (right). The simulated virtual image in the conventional system covers only part of the numbers 4–6, namely, a 13° FOV, consistent with Eq. (1). In contrast, the experimental results in Fig. 4c demonstrate that the proposed light-field NED covers numbers 1–9 over a 50° FOV. The incorporation of a beam splitter enables simultaneous viewing of virtual and physical world information. The measured 50° FOV matches the simulation results in Fig. 4a (bottom). Additionally, as shown in the magnified view in Fig. 4c, the distinguishable pixel contours indicate that the MeLA resolution meets the micro-display’s pixel-size requirements. The vertical FOV limit is also tested, presented in Fig. 4d. Since no FOV-expanded design is implemented here, the FOV remains approximately 12°, consistent with the result in Fig. 4b. By comparing the horizontal and vertical FOVs, our FOV-expanded light-field NED achieves approximately four times the FOV compared to the conventional architecture. Figure 4d also shows that the discretely arranged MeLA enables continuous display across the entire FOV. The faint replicated images vertically adjacent to the primary image in Fig. 4d come from crosstalk, which occurs when an elemental image reaches the eyebox through the lenslet adjacent to the correct one. In the lateral direction, due to the heterogeneous design of the MeLA, elemental images are accurately guided to the eyebox without passing through adjacent lenslets, resulting in little crosstalk, particularly at large FOV angles. On the other hand, no FOV expansion was performed in the vertical direction; accordingly, the crosstalk issue was not addressed. We prototyped wearable XR glasses using 3D printing (see Fig. S10 in Supplementary Information), envisioning a possible application scenario of 3D levitation navigation. By integrating virtual navigation information with real scene information, a precise and vivid navigation effect can be achieved. Figure 4e shows the meta-based light-field NED prototype in the corridor outside the laboratory. The virtual navigation information is displayed in the middle of the FOV at approximately 25 cm (the standard distance for distinct vision), indicating the need to turn right after 3 m.

**Fig. 5:**
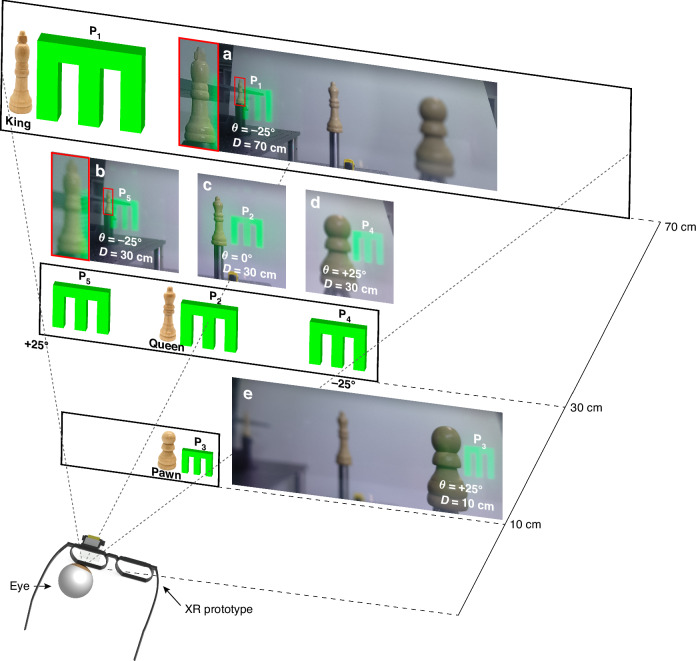
. **Demonstration of monocular 3D display capability**. Three chess pieces serve as depth markers positioned at vertical distances of 70 cm, 30 cm, and 10 cm from the camera. The camera remains focused on the character “E”, where D denotes the current vertical distance between the character “E” and the camera, θ denotes the azimuth angle of the current position of “E”. **a** The “E” is at position P_1_, in the same depth plane as the farthest chess piece “King”. Note that “E” and the chess piece “King” are clear, while the intermediate and the nearest chess pieces are blurred. **b**–**d** The “E” is at positions P_5_, P_2,_ and P_4,_ respectively, in the same depth plane with the intermediate chess piece “Queen”. Note that “E” and the chess piece “Queen” are clear, while the farthest and the nearest chess pieces are blurred. **e** The “E” is at position P_3_, in the same depth plane as the nearest chess piece, “Pawn”. Note that “E” and the nearest chess piece, “Pawn,” are clear, while the farthest and the intermediate chess pieces are blurred

The proposed FOV-expanded light-field NED supports VAC-free 3D. As shown in Fig. 5, we chose the character “E” facing downward as the virtual image to move continuously. Three chess pieces are used as depth markers, fixed respectively at the vertical distances of 70, 30, and 10 cm from the camera (as the human eye). P_1_ is 70 cm from the camera at −25° of the FOV. P_2_, P_4_, and P_5_ are positioned 30 cm vertically from the camera at 0°, +25°, and −25°, respectively. P_3_ is 10 cm vertically from the camera at +25°. The virtual characters have two motion trajectories: (i) P_1_, P_2_, then to P_3_, and (ii) P_4_, P_2_, then to P_5_. During the motion, the camera continuously focuses on the “E”. As shown in Fig. 5a, when “E” is at position P_1_, both “E” and the farthest chess piece, “King,” are clear, while the intermediate and nearest chess pieces are relatively blurred. In Fig. 5b–d, when “E” is at positions P_5_, P_2_, and P_4,_ respectively, “E” and the intermediate chess piece “Queen” are clear, while the farthest and nearest chess pieces are blurred due to defocus. In Fig. 5e, when “E” is at P_3_, “E” and the closest “Pawn” are clear, while the intermediate and farthest chess pieces are blurred. The related motion videos are available in Movies S1 and S2 in Supplementary Information.

Hence, the proposed FOV-expanded light-field NED simultaneously provides a 3D reconstruction depth range of approximately 60 cm and a horizontal FOV of 50°. It can present realistic visual effects in a monocular view. Additionally, a virtual object can utilize the same depth cues as real objects to blend seamlessly with the real world. The characteristics of a wide FOV and VAC-free have potential applications in ophthalmology and are expected to assist in the exercise of eye muscles for self-correction of strabismus^55^.

## Discussion

The proposed FOV-expanded light-field NED has a FOV rivaling current AR products, yet this is not the limit of the above experimental results. According to Eq. (2), the FOV is eventually determined by the micro-display size, so a larger FOV is achievable with a larger micro-display. In Section 2 of Supplementary Information, we simulated a micro-display of 30.8 mm paired with a MeLA of the same size. As a result, an 86° FOV is achieved, exceeding the entire range of stereopsis. Furthermore, this FOV expansion scheme can be readily extended to two axes. A simulation of a design achieving a symmetrical 50° × 50° FOV is provided in Supplementary Information, Section 8. As NED technology advances, more display manufacturers are expected to introduce larger-sized and higher PPI micro-displays, which can unlock more significant potential for this unique meta-based FOV extension scheme.

Alongside FOV, the eyebox is another critical parameter that defines the tolerance for eye movement. In our current prototype, the static eyebox is approximately 4 mm in diameter, determined by the exit pupil of a single lenslet. While this provides a functional viewing zone, we envision a pathway to a larger, more user-friendly eyebox by integrating eye-tracking. This strategy, successfully demonstrated in other NED architectures^56^, would create a virtually expanded dynamic eyebox without compromising the FOV. Our elemental image array generation algorithm is inherently well-suited for the real-time pattern adjustments required by such a dynamic scheme.

We intentionally designed the MeLA with a discrete rather than a fully filled column layout for several reasons. Each metalens column provides a FOV of approximately 12°. In a fully filled configuration, the FOVs of adjacent columns overlap significantly. Consequently, strategically omitting specific columns does not compromise the overall expanded FOV. To validate this, we performed simulations confirming that there is no significant difference in the final FOV expansion between a fully filled and a discrete MeLA (see Supplementary Information, Section 9).

Furthermore, as shown in Fig. 4d, a carefully engineered discrete MeLA can still produce a seamless and continuous visual field. The primary advantage of this discrete design is the substantial reduction in both the fabrication time and cost of the nanoimprint master mold, while still achieving the core objectives of this work. However, the design is not without a trade-off. Due to the wide emission angle of the commercial micro-display, a portion of light passes through the blank areas between columns without being modulated by the array. This unmodulated light may enter the eyebox, becoming stray light that degrades image contrast, as observed in Figs. 4 and 5. Despite these challenges, our design demonstrates considerable robustness against typical fabrication imperfections. A detailed tolerance analysis, presented in Supplementary Information Section 10, shows that reasonable variations in nanopillar dimensions and the presence of minor defects have a manageable impact on the final imaging quality.

Building upon our prior work that pioneered VAC-free NEDs using a homogeneous MeLA^26^, this paper introduces a significant conceptual and technological leap forward. We identified and addressed the key limitation of our previous approach: the narrow FOV caused by off-axis aberrations. The heterogeneous MeLA, functioning as a lens cascading prism, was proposed to address the issue of narrow FOV. This new design paradigm, which decomposes a complex off-axis imaging problem into simple on-axis imaging and deflection tasks, represents a fundamental departure from conventional thinking and enables the simultaneous achievement of a 3D VAC-free display with a wide FOV. The superiority of metalenses in terms of wavefront control flexibility and extreme thinness is fully leveraged by elaborating each lenslet in the MeLA to control the FOV independently.

To contextualize our contribution, a detailed comparison with other state-of-the-art near-eye displays is provided in Table S1 of the Supplementary Information. The comparison highlights the unique position of our work. While each technology excels in certain aspects, such as a wide FOV from a curved microlens array or an achromatic metalens eyepiece, our heterogeneous metalens array is distinct in its ability to simultaneously provide a 3D VAC-free experience and a wide FOV within a single, ultra-thin, planar component. This specific combination of features provides a promising way to XR displays.

In conclusion, we analyzed the FOV limitation of MeLA light-field NED. The MeLA is fabricated via nanoimprint, overcoming the challenges of large-scale fabrication. The nanostructures are composed of high-n imprint material, with a maximum aspect ratio of 7:1. By developing a fast elemental image array generation algorithm dedicated to the MeLA, a FOV-expanded light-field NED prototype was built. A FOV of 50° has been experimentally achieved, four times that of conventional systems, without compromising other performance or increasing the system volume. The maximum FOV limit supported by commercial micro-displays has been fully exploited. We also verified a depth range of 60 cm to demonstrate the effectiveness of monocular depth cues. While the current demonstration is monochromatic, the pathway to extend our heterogeneous design to full-color, achromatic operation is further discussed in Supplementary Information (Section 11). The proposed FOV-expanded light-field NED is expected to address the critical challenge of achieving wide FOV and VAC-free 3D for XR displays.

## Materials and methods

The display simulation in this paper uses ray tracing to simulate how the human eye perceives the display, based on the architecture of “pattern light source—MeLA—receiver.” Specifically, the patterned light source comprises a backlight and an elemental image array mask. At the same time, the receiver acquires optical data and, in turn, renders virtual images at designated depth planes via light propagation trajectories.

The elemental image array generation algorithm was implemented on a cost-effective laptop (MECHREVO Forza 14; AMD Ryzen 7-7840HS; integrated graphics) using MATLAB. By integrating an elemental image array rendering correction algorithm with a lookup table-based acceleration strategy, the system achieves an average rendering time of 7 ms per frame.

The experiment utilized a Sony ECX335SN microdisplay with a 3135 PPI (pixel pitch: 8 microns).

## Supplementary information

Supplementary Information for Expanded field of view light-field extended-reality displays with metalens array

Experimental movie S1

Experimental movie S2

## Data Availability

All data needed to evaluate the conclusions in the paper are present in the paper and/or the Supplementary Information. Additional data related to this paper may be requested from the corresponding authors.
