# Supplementary material for: Expanded field of view light-field extended-reality displays with metalens array
Source: Light Sci Appl. 2026 Jun 29;15:293. doi: 10.1038/s41377-026-02266-w (PMC13315926; doi:10.1038/s41377-026-02266-w)
Supplement: Supplementary file 1 — Supplementary Information for Expanded field of view light-field extended-reality displays with metalens array [file 41377_2026_2266_MOESM1_ESM.docx]

Supplementary Information for

**Expanded field of view light-field extended-reality displays with metalens array**

Wen-Long Lu^1,2^, Yang Yi^1,2^, Zhi-Bin Fan^1,2^, Shi-Hao Li^1,2^, Zong Qin^2,3,4^*, Jian-Wen Dong^1,2^*

**Affiliations：**

^1^School of Physics, Sun Yat-sen University, Guangzhou 510275, China.

^2^State Key Laboratory of Optoelectronic Materials and Technologies, Sun Yat-sen University, Guangzhou 510275, China.

^3^School of Electronics and Information Technology, Sun Yat-sen University, 132 East Waihuan Rd, Guangzhou 510006, China.

^4^Guangdong Province Key Laboratory of Display Material and Technology, Sun Yat-sen University, 132 East Waihuan Rd, Guangzhou, 510006, China.

*Corresponding authors, Email: [qinzong@mail.sysu.edu.cn](mailto:qinzong@mail.sysu.edu.cn), [dongjwen@mail.sysu.edu.cn](mailto:dongjwen@mail.sysu.edu.cn)

**This PDF file includes:**

Section 1. Field of view expansion for integral imaging light-field near-eye displays

Section 2. FOV simulation of the light-field NED based on a large-scale MeLA

Section 3. Simulation results of nanopillar grating

Section 4. Fabrication of the MeLA nanoimprint master mold

Section 5. Fabrication of MeLA by nanoimprinting

Section 6. Performance characterization of the MeLA

Section 7. Elemental image array rendering correction algorithm

Section 8. Two-axis FOV expansion

Section 9. FOV simulation of the light-field NED based on the fully filled MeLA

Section 10. Fabrication tolerance analysis

Section 11. Fabrication trade-offs and pathway to an achromatic design

Fig. S1. Comparative analysis of FOV expansion schemes in light-field NEDs

Fig. S2. FOV simulation of the light-field NED based on large-scale MeLA

Fig. S3. Simulation results of the nanopillar grating at 525 nm wavelength

Fig. S4. Simulated phase and transmission maps of the meta-atoms at 525 nm wavelength

Fig. S5. Schematic flowchart of the EBL process for fabricating the master mold

Fig. S6. The fabricated MeLA nanoimprint silicon master mold

Fig. S7. Schematic diagram of the MeLA nanoimprint process

Fig. S8. Performance characterization of the fabricated MeLA

Fig. S9. Schematic diagram illustrating the nonlinear pixel-voxel mapping relationship in meta-based light-field NED

Fig. S10. The wearable meta-based XR glasses prototype

Fig. S11. Design and simulation of a two-axis FOV expansion.

Fig. S12. Simulation of the FOV for light-field NED based on the fully filled MeLA

Fig. S13. Simulation of fabrication tolerance

Fig. S14. Simulated full-color imaging performance of a future achromatic heterogeneous MeLA

Table S1 Comparison of near-eye display technologies

Movie S1. The virtual character "E" traverses from distant to proximal positions

Movie S2. The virtual character "E" traverses from the right to the left side.

**Section 1. Field of view expansion for integral imaging light-field near-eye displays**

In this section, we introduce the conventional method for calculating the field of view (FOV) of an integral-imaging light-field near-eye display (NED) and propose three expanded-FOV schemes for comparison. By default, the microlens array has the same size *S* as the microdisplay.

**Conventional light-field NED**

Generally, when elemental images are modulated only by the microlens unit directly below them and then enter the human eye, as shown in Fig. S1a, the maximum FOV is expressed as:

$FOV_{0}=2\cdot\arctan(\frac{w}{2g})$ (1)

where *w* = 400 μm is the lenslet pitch, *g* = 1.8 mm is the gap between the microlens array and the micro-display, and eye relief *r* = 18 mm. The effective display area of the micro-display is *S*_0_, satisfying *S*_0_ ≤ (1 + *r*/*g*)*w*. In this case, a qualified microlens array can typically maintain near-diffraction-limited imaging within *FOV*_0_, but *FOV*_0_ is very narrow, approximately 13°. Moreover, *S*_0_ is only 4.4 mm, far from fully utilizing the selected micro-display.

**FOV expansion scheme 1**

In this scheme, elemental images can be modulated by adjacent microlens lenslets and then enter the human eye, as shown in Fig. S1b. The maximum FOV can be expanded as:

$FOV_{1}=2\arctan(\frac{S_{1}}{2(r+g\text{)}})$ (2)

The effective display area of the micro-display is *S*_1_. This scheme can achieve a larger FOV and fully utilize the screen’s size. For example, when the effective display area *S*_1_ = *S* = 13.2 mm, *FOV*_1_ is approximately 37°. Compared with the conventional scheme, the FOV is expanded by three times. However, the large off-axis angle of elemental images at the edges may cause severe aberrations and brightness attenuation, significantly degrading edge display quality. Moreover, some elemental images can enter the human eye through both the microlens unit directly above them and adjacent microlens lenslets, which may lead to image crosstalk and stray light problems.

**FOV expansion scheme 2**

To address the issue in Scheme 1 where severe aberrations are introduced to expand the FOV, the microprism units with varying deflection angles can be superimposed on the microlens units to form a spatially heterogeneous microlens array with composite units, as shown in Fig. S1c. This scheme ensures that the elemental image area modulated by the microlens unit is always directly above it, thereby avoiding severe off-axis aberrations and image crosstalk. Assuming the deflection angle of the *i*-th microprism unit is *α_i_*. The light from the edge pixels of the elemental image is incident at an angle *θ* to the center of the composite unit below. After being modulated by the composite unit, it enters the pupil center at an angle *β_i_*, satisfying the following Eq. (3):

$\begin{matrix} \sin\alpha_{i}=\sin\beta_{i}-\sin\theta\\ \text{with }\beta_{i}=\arctan\left(\frac{x_{ic}}{r}\right),\theta\text{=}\arctan\left(\frac{w}{2g}\right) \end{matrix}$ (3)

where *x_ic_* denotes the horizontal coordinates of the center of the *i*-th microlens unit. At this time, the deflection angle *α_i_* of the *i*-th microprism unit is expressed as:

$\alpha_{i}=\arcsin\left(\sin\left(\arctan\left(\frac{x_{ic}}{r}\right)\right)-\sin\left(\arctan\left(\frac{w}{2g}\right)\right)\right)$ (4)

The maximum *FOV*_2_ is:

$FOV_{2}=2\beta_{\max}=2\arctan\left(\frac{S_{2}-w}{2r}\right)$ (5)

When the effective display area size *S*_2_ = S = 13.2 mm, *FOV*_2_ is approximately 39°. This scheme not only offers the advantages of conventional near-diffraction-limited imaging but also provides a FOV comparable to that of Scheme 1.

**FOV expansion scheme 3**

In Scheme 2, we let the light from the edge pixels of the micro-display pass through the composite unit and enter the center of the pupil. However, in reality, the pupil is not a point but a range with a width of *P*. As long as the light enters this range, it can be observed. Therefore, we can adjust the design of the deflection angle *α_i_* of the microprism unit so that the light from the pixel directly above the center of the microlens unit is deflected to the exact center of the pupil after modulation. At this point, the deflection angle *α_i_* of the *i*-th microprism unit can be calculated by the following equation:

$\alpha_{i}=\arctan\left(\frac{x_{ic}}{r}\right)$ (6)

The deflection angle *α*_max_ of the most edge microprism unit is:

$\alpha_{\max}=\arctan(\frac{S_{3}-w}{2r})$ (7)

And the angle *β*_max_ of the light from the most edge pixel of the micro-display after being modulated by the composite unit satisfies:

$\sin\beta_{\max}=\sin\alpha_{\max}+\sin\theta$ (8)

The FOV range that the micro-display can provide is:

$\begin{matrix} FOV_{\text{S}}=2\beta_{\text{max}}=2\text{arcsin}\left(\sin\theta+\sin\alpha_{\text{max}}\right) \\ =2\text{arcsin}\left(\text{sin}\left(\text{arctan}\left(\frac{w}{2g}\right)\right)+\text{sin}\left(\text{arctan}\left(\frac{S_{3}-w}{2r}\right)\right)\right) \end{matrix}$ (9)

However, the edge light may exceed the pupil range. If only the light falling within the pupil is considered, the FOV can be calculated by reverse tracing the light at the edge of the pupil, as shown in Fig. S1d. Hence, the system's FOV becomes:

$FOV_{\text{P}}=2\arctan(\frac{S_{3}-w+P}{2r})$ (10)

The maximum FOV of the system in this scheme depends on the minimum value of Eqs. (9) and (10), that is, FOV = min(*FOV*_S_, *FOV*_P_). When the pupil width is fixed at *P* = 4 mm, *S*_3_ = 13.2 mm, *w* = 400 μm, *g* = 1.8 mm, and *r* = 18 mm, there is *FOV*_S_ = 53° > *FOV*_P_ = 50°. As a result, the system’s FOV can be expressed by Eq. (10), reaching 50°, which is about four times that of the conventional scheme and 11° larger than the FOV of Scheme 2.

**Section 2. FOV simulation of the light-field NED based on a large-scale MeLA**

As mentioned in the main text, a 50° FOV is not the limit of our meta-based FOV extension scheme. The size of the commercial micro-display limits it, and also the size of the heterogeneous metalens array (MeLA) as a light control device. Here, we assume the screen PPI remains constant as the display size increases, and the display area reaches 30.8 mm in the horizontal dimension. We designed a heterogeneous MeLA with a width of 30.8 mm, composed of a 77 × 2 array of metalenses, with each column having a different deflection angle (*α_i_*). In contrast, the width and focal length of each metalens remained constant. Simulations incorporating the elemental image array rendering correction algorithm (Section 7) demonstrate that a high-quality display effect is maintained across a horizontal FOV of approximately 86° (Fig. S2). This result highlights the significant potential of our proposed metalens-based approach for FOV expansion.

**Section 3. Simulation results of nanopillar grating**

The phase and transmittance versus diameter curves of nanopillar gratings, normalized to different conditions at 525 nm, are shown in Fig. S3. In Fig. S3a, the nanopillar unit of the MeLA in this paper is shown with a lattice period of L = 330 nm on a SiO_2_ substrate. The nanopillars grating's phase and transmittance curves as functions of diameter for each of the five deflection angles (α) in the MeLA are shown in Figs. S3b-f. The diameter range of the nanopillars is 100-230 nm, and for ease of comparison, the phase response of the 100 nm diameter is set to zero. In Fig. S3b, when the refractive index n = 1.5 and height h=700 nm, the phase coverage is only 0.46π. For n = 1.9 and h = 500 nm, the phase coverage increases to 0.73π. When n = 1.9 and h = 700 nm, the phase coverage reaches 1.02π. The transmittance levels remain comparable across all three cases. Comparison of the three cases reveals that the higher the refractive index and the taller the nanopillar, the greater the phase interval coverage. The transmittance in the three cases does not differ significantly from each other. From Figs. S3b-f, one can find that the same conclusions can be drawn at five angles of incidence.

To further characterize the fundamental properties of our meta-atoms, we simulated the phase and transmission response of the cylindrical nanopillars as a function of both incident angle and diameter. The results, calculated for both S- and P-polarizations, are presented in Figure S4. A comparison between the S-polarization (Fig. S4a) and P-polarization (Fig. S4b) results reveals the polarization response of our design. The overall trends in both phase and transmission are highly similar across the two polarization states, particularly for incident angles below approximately 20°. In this range, the design exhibits a very low degree of polarization sensitivity. Since the maximum design deflection angle (*α*) used in our heterogeneous MeLA is less than 20°, our design operates entirely within this polarization-insensitive regime. While noticeable differences between the two polarizations begin to emerge at larger incident angles (>20°), this is outside our operational range. Therefore, the robust and polarization-insensitive performance across our full range of design angles is highly beneficial for applications involving unpolarized light sources, such as micro-OLEDs.

**Section 4. Fabrication of the MeLA nanoimprint master mold**

As shown in Fig. S5, the 4-inch silicon wafer was first thoroughly cleaned. Second, the negative electron-beam resist (Hydrogen silsesquioxane, HSQ) was spin-coated onto the silicon wafer surface. Then, the MeLA pattern was exposed in a high-resolution HSQ film using an electron-beam lithography (EBL) system (EBPG5000 ES, Vistec) operating at 100 kV. After exposure, the development is performed in tetramethylammonium hydroxide (TMAH). Next, inductively coupled plasma etching (PlasmaPro System100 ICP180, Oxford Instruments) using hydrogen bromide (HBr) gas was performed to achieve a depth of 700 nm. Finally, the residual resist was removed by wet etching with hydrofluoric acid (HF) solution, yielding a silicon master mold, as shown in Fig. S6a, where the red rectangular frame highlights the nanostructured area. Fig. S6b is the microscopic image of the silicon master mold obtained by stitching microscope images, and Fig. S6c shows a top-view SEM image of a partial region of the silicon master mold.

**Section 5. Fabrication of MeLA by nanoimprinting**

As mentioned in the main text, imprinting high-refractive-index, high-aspect-ratio nanostructures is challenging. When fabricating the MeLA samples (refractive index = 1.9, maximum aspect ratio = 7:1) using the nanoimprint method, bubble-like defects were observed. To address this defect, we improved the nanoimprint process. As shown in Fig. S7a, the silicon master mold is first treated with an anti-sticking process to add a hydrophobic layer on the surface. The contact angle of water droplets after the anti-sticking treatment should be greater than 90° to prevent difficulties in demolding the stamp in the subsequent process. Then, in Fig. S7b, the stamp material is spin-coated at high speed onto the master mold to ensure it fills the master mold’s gaps and maintains a flat upper surface. After that, in Fig. S7c, a transparent PET film is used as the carrier for the stamp, which is pressed onto the stamp material under a certain pressure, and then ultraviolet exposure curing is initiated. Then, the stamp material is bonded to the PET carrier, and the stamp can be obtained after demolding in Fig. S7d. Liquid imprint material is dropped onto the stamp, and vibration is applied to ensure that the imprint material fully fills the stamp and expels air, thus ensuring that the final sample has no bubble-like defects. Then, the excess imprint material is spin-coated off in Fig. S7e, and the parameters are carefully adjusted to control the thickness and flatness of the upper surface of the imprint material. After high-temperature baking, the excess solvent in the imprint material is removed.

Meanwhile, the cleaned SiO₂ substrate underwent adhesion promotion treatment for subsequent use. Then, in Fig. S7f, the stamp with the imprint material is pressed onto the treated SiO_2_ substrate with a certain pressure, and ultraviolet exposure begins. After exposure, demolding starts, and the demolding angle and speed parameters are carefully adjusted to ensure that the nanostructures are smoothly transferred to the substrate, as shown in Fig. S7g.

**Section 6. Performance characterization of the MeLA**

Here, we provide additional details not mentioned in the main text. The light-field scanning system is shown in Fig. 3a of the main text. The laser is emitted from a supercontinuum laser (WL-SC480-20-PP, Fianium) and filtered through a narrow-band filter (LLTF-VIS2-HP-FDS-SM, Fianium). Then, the light intensity is adjusted by an attenuator (GCO-07M, DHC). Since the laser spot is too small to uniformly cover the MeLA lenslet, a beam expander (GCO-2503, DHC) is used to expand the spot size, ensuring that the incident light is uniformly illuminated on the MeLA lenslet during the test. All these components together form the incident light system, which is mounted on an electrically controlled rotary displacement stage (MRS103, BOCIC) to precisely control the angle of incident light on the MeLA lenslet. The MeLA is placed on a high-precision, electrically controlled translation stage (9063-XYZ-PPP-M, Newport) for scanning the light-field distribution. The MeLA surface is set to z = 0, and the light propagates in the positive z direction. The microscopic imaging system behind the MeLA consists of a 20× objective lens (MPlanFLN20xBD, Olympus), a tube lens (Thorlabs ITL200), and a CCD detector (HAMAMATSU C11440-22CU). A calibration ruler in front measures the magnification between the CCD image pixels and the actual physical scale. By moving the sample along the z-direction using the electric translation stage, the surface and focal plane of the MeLA lenslet can be identified. By scanning light-field intensity images at different z-planes, the propagation field-intensity distribution of the MeLA can be obtained.

In Figs. 3b-e of the main text, only the test results of five types of MeLA lenslets in the array are listed. Here, we supplement the measurement results of the other four types of MeLA lenslets as shown in Figs. S8a-d, including the normalized light field intensity distributions in the *x-z* plane, the normalized light field intensity distributions at the focal plane, the focal plane profile curves, and the corresponding MTFs. In addition, the intensity profile curves of the focal spots of the nine types of MeLA units along the *x*-direction and their corresponding MTFs are shown in Figs. S8e-f. It can be observed from the figure that the full width at half maximum (FWHM) of the *x*-direction focal spot profiles increases with the deflection angle *α*, ranging from 2.1 to 3.4 μm, which is much smaller than the pixel size of the microdisplay (~8 μm).

**Section 7. Elemental image array rendering correction algorithm**

In traditional elemental image array generation algorithms, the mapping between pixels and voxels follows a simple linear relationship, with the lateral magnification between them on the same reconstruction depth plane remaining constant. As mentioned in the main text, we propose a heterogeneous MeLA. The deflection effect of our MeLA ensures that all element images fall in the paraxial region directly above the MeLA units during acquisition. As shown in Fig. S9, this deflection effect can be represented by the following mapping relation:

 (11)

where *d* denotes the distance from the rebuilt depth plane to the MeLA, *x_i_* denotes the pixel's distance perpendicular to the optical axis, *L* denotes *x_i_*’s projection on the rebuilt depth plane, *ζ* denotes the angle between the main light ray emitted from the pixel point and the optical axis, *α_i_* denotes the unit's deflection angle and *φ* denotes the angle between the light ray deflected through the MeLA unit and the optical axis. Unlike the simple linear mapping relationship (*L* = *x_i_* ∙*d*/*g*) between pixels and voxels in traditional elemental image array generation algorithms, Eq. (11) shows that under the influence of the prismatic phase in MeLA, the pixel-voxel mapping transitions to a nonlinear relationship. Based on the mapping relationship, we can derive the transverse magnification *γ*:

$\gamma(x_{i})=\frac{\partial L}{\partial x_{i}}=dg^{2}\cdot\frac{1}{(1-(\sin\alpha_{i}\text{+}\frac{x_{i}}{\sqrt{g^{2}+x_{i}^{2}}})^{2})^{\frac{3}{2}}}\cdot\frac{1}{\left(g^{2}+x_{i}^{2}\right)^{\frac{3}{2}}}$ (12)

Eq. (12) shows that the transverse magnification of a pixel under a single MeLA unit at the same rebuilt depth plane is no longer a fixed value, but a function of *α_i_* and *x_i_*. Based on Eqs. (11) and (12), we propose an elemental image array rendering correction algorithm applicable to meta-based light-field NED. This algorithm can utilize pre-correction processing to compensate for changes in transverse magnification.

The correction algorithm includes the following steps:

(i) Voxel plane sampling. The input 3D scene or image is sampled according to the voxel size, which is determined by the minimum projection size of a pixel onto the voxel plane.

(ii) Drawing voxel projections. Based on the transverse magnification distribution function and the coordinate mapping function, the position and shape of the voxel projection on the pixel plane are calculated, and the projection map of the voxel plane is drawn.

(iii) Pixel plane sampling. The projection map is resampled by the pixel plane, and the elemental image array is output.

**Section 8. Two-axis FOV expansion**

The design framework presented in the main text is inherently scalable to achieve two-axis FOV expansion. The generalized phase profile for a metalenslet at position (*i*, *j*) within such an array can be expressed as:

 (13)

Compared to Eq. (3) in the main text for horizontal FOV expansion, this profile incorporates an additional linear phase tilt in the vertical direction (y-term) to deflect the beam. The deflection angles *αᵢ* and *α_j_* for each lenslet are determined by its center coordinates (*x_ijc_*, *y_ijc_*) and the designed eye relief *r.* To demonstrate the feasibility of this two-axis expansion, we designed a heterogeneous metalens array where the deflection angles *αᵢ* and *αⱼ* were distributed across a 2D grid, as illustrated in Fig. S11a. The array was then simulated in our light-field near-eye display configuration. Fig. S11b shows the simulation result for this design. As shown, the system successfully achieves a large, continuous, and symmetrical 50° × 50° FOV, confirming that our proposed method is fully capable of delivering symmetrical wide-FOV performance and provides a clear pathway to highly immersive 3D near-eye displays.

**Section 9. FOV simulation of the light-field NED based on the fully filled MeLA**

Here, we demonstrate the FOV simulation results of the light-field NED based on the fully filled MeLA. As shown in Fig. S12a, the fully filled MeLA consists of 33 columns and eight rows of lenslets, with each column having a different deflection angle, *α*. The discrete MeLA used in the main text retains only nine columns. Fig. S12b shows the FOV simulation results of the light-field NED based on the fully filled MeLA, with a horizontal FOV of 50°. Compared with the simulation results in Fig. 4a (bottom) of the main text, there is no significant difference in the FOV range.

**Section 10. Fabrication tolerance analysis**

To assess the robustness of our heterogeneous metalens array against practical manufacturing imperfections, we performed a series of numerical simulations. This analysis is crucial for evaluating the design's feasibility for scalable fabrication methods like nanoimprint lithography, where minor dimensional deviations and defects are inevitable. Our methodology involves simulating the Point Spread Function (PSF) for metalenses with predefined fabrication errors, convolving this aberrated PSF with a target image, and quantitatively evaluating the output quality using two standard metrics: the Peak Signal-to-Noise Ratio (PSNR) and the Structural Similarity Index Measure (SSIM), with the image from an ideal, error-free metalens serving as the benchmark.

We investigated several representative fabrication deviations, as presented in Fig. S13. These include systematic variations in nanopillar diameter (±20 nm) and height (±20 nm). It is important to note that these simulated tolerance ranges represent a conservative, worst-case scenario; our actual fabrication process can control diameter and height variations to well within ±20 nm. Additionally, we simulated random defects by assigning random amplitude and phase responses to 10% of the nanopillars, mimicking potential imprint filling or residue issues. The simulation results demonstrate that even under these conservative error conditions, the overall impact on image quality is manageable, as indicated by the high PSNR and SSIM values. This analysis confirms that our design exhibits significant tolerance to typical, achievable fabrication deviations, reinforcing its potential for practical implementation.

**Section 11. Fabrication trade-offs and pathway to an achromatic design**

The decision to focus on a monochromatic design in the main text is rooted in current fabrication trade-offs. Achieving both large-angle aberration correction and broadband achromatism places extreme demands on the fabrication process. In nanoimprint lithography, this means either ultra-high aspect ratios or more complex nanostructures are required. While our current aspect ratio of 7:1 for a high-refractive-index polymer(n = 1.91 @ 525nm) is at the forefront of scalable fabrication, it is still insufficient for this complex, dual-objective optimization. Nevertheless, a clear path to achromatization exists. We envision two primary strategies for future work.

1. Process advancement: Pushing the limits of nanoimprint technology to achieve ultra-high aspect ratios (e.g., >30:1) with simple nanostructures, which can provide the design space for dispersion engineering.
2. Advanced fabrication & design: adopting more complex nanostructure geometries, similar to prior work in (ref. 40). This would necessitate a shift towards low-throughput, high-precision fabrication methods compatible with higher-refractive-index materials, such as E-beam lithography followed by etching, trading off manufacturing scalability for ultimate performance.

To illustrate the ultimate potential of such an achromatic approach, Fig. S14 presents a simulated full-color imaging result. Fig. S14b was generated by convolving a target image (Fig. S14a) with a set of experimentally measured full-color PSFs obtained from the achromatic metalens work (ref. 40). This simulation serves as a conceptual benchmark, demonstrating that the high-fidelity color performance is achievable when our heterogeneous design paradigm is combined with a proven achromatic design.


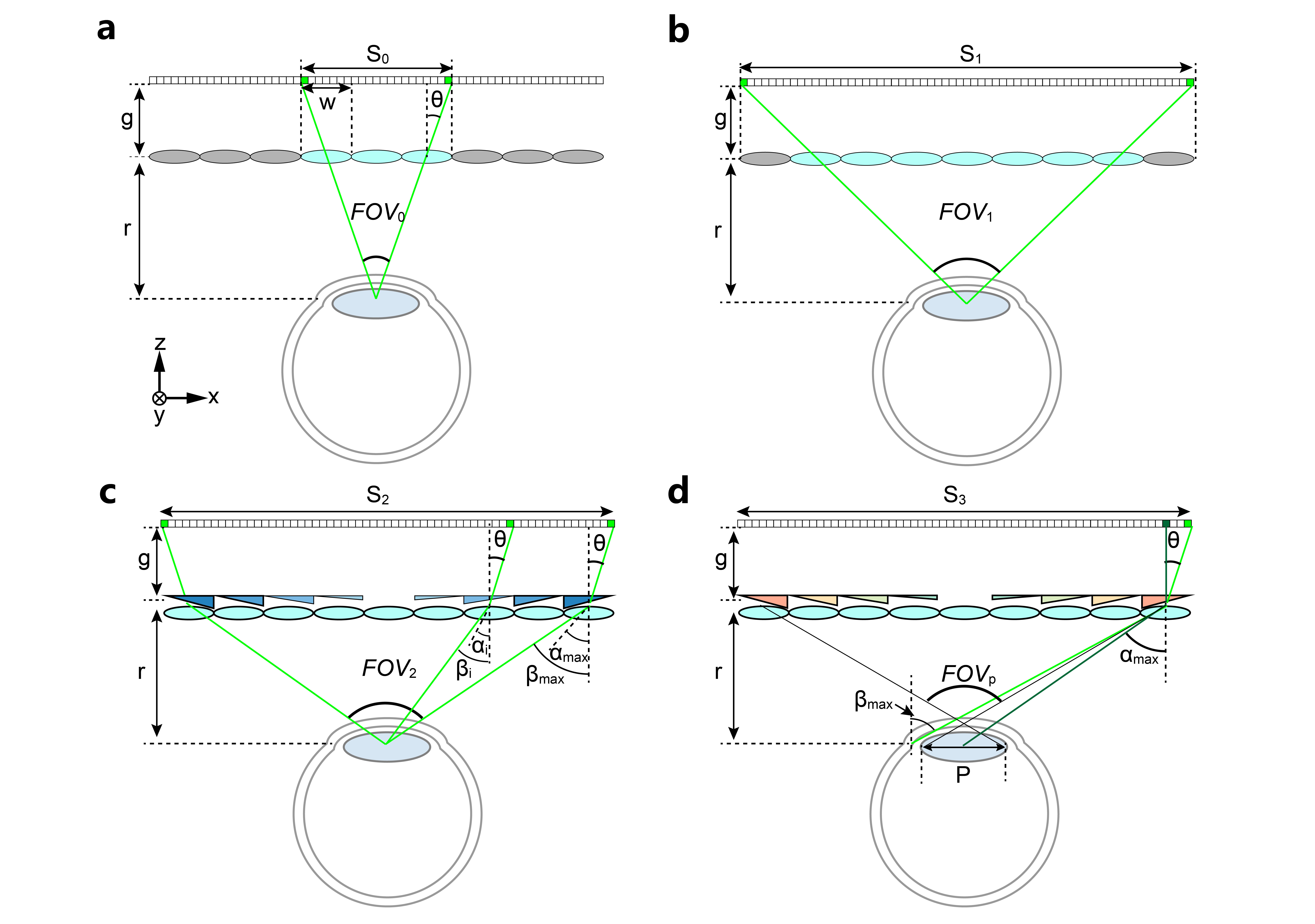


**Fig. S1. Comparative analysis of FOV expansion schemes in light-field NEDs.** **a** FOV schematic of the conventional light-field NED. **b** FOV expansion scheme 1 achieves FOV enlargement but suffers from severe off-axis aberrations in peripheral FOV regions. **c** Scheme 2 employs a spatially heterogeneous microlens array to address edge degradation in Scheme 1. **d** Scheme 3 optimizes prism deflection angles in the heterogeneous array compared to Scheme 2, further expanding the FOV and attaining maximum FOV within the system's configuration limits.


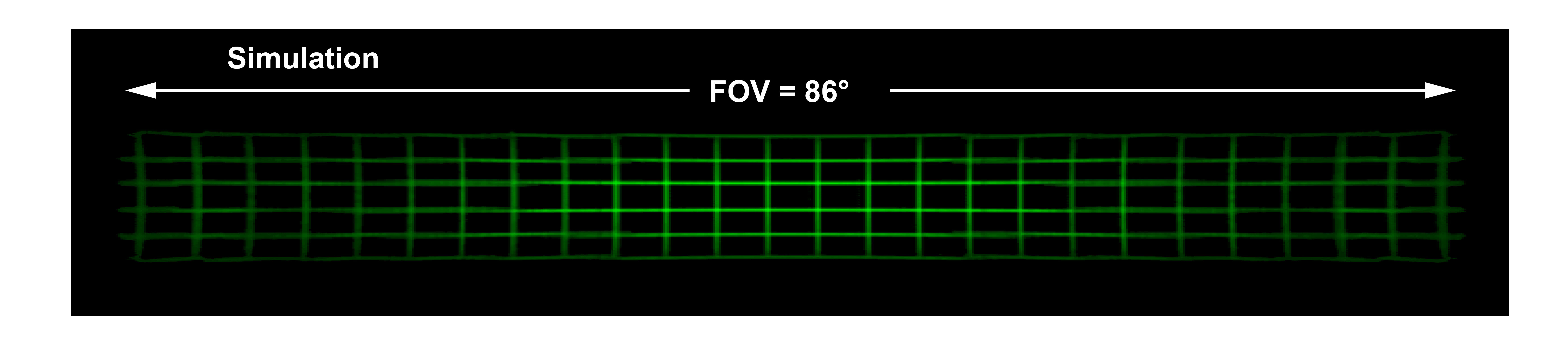


**Fig. S2. FOV simulation of the light-field NED based on a large-scale MeLA.** The simulation shows a horizontal FOV of 86°, consistent with the theoretical prediction from Eq. 10.


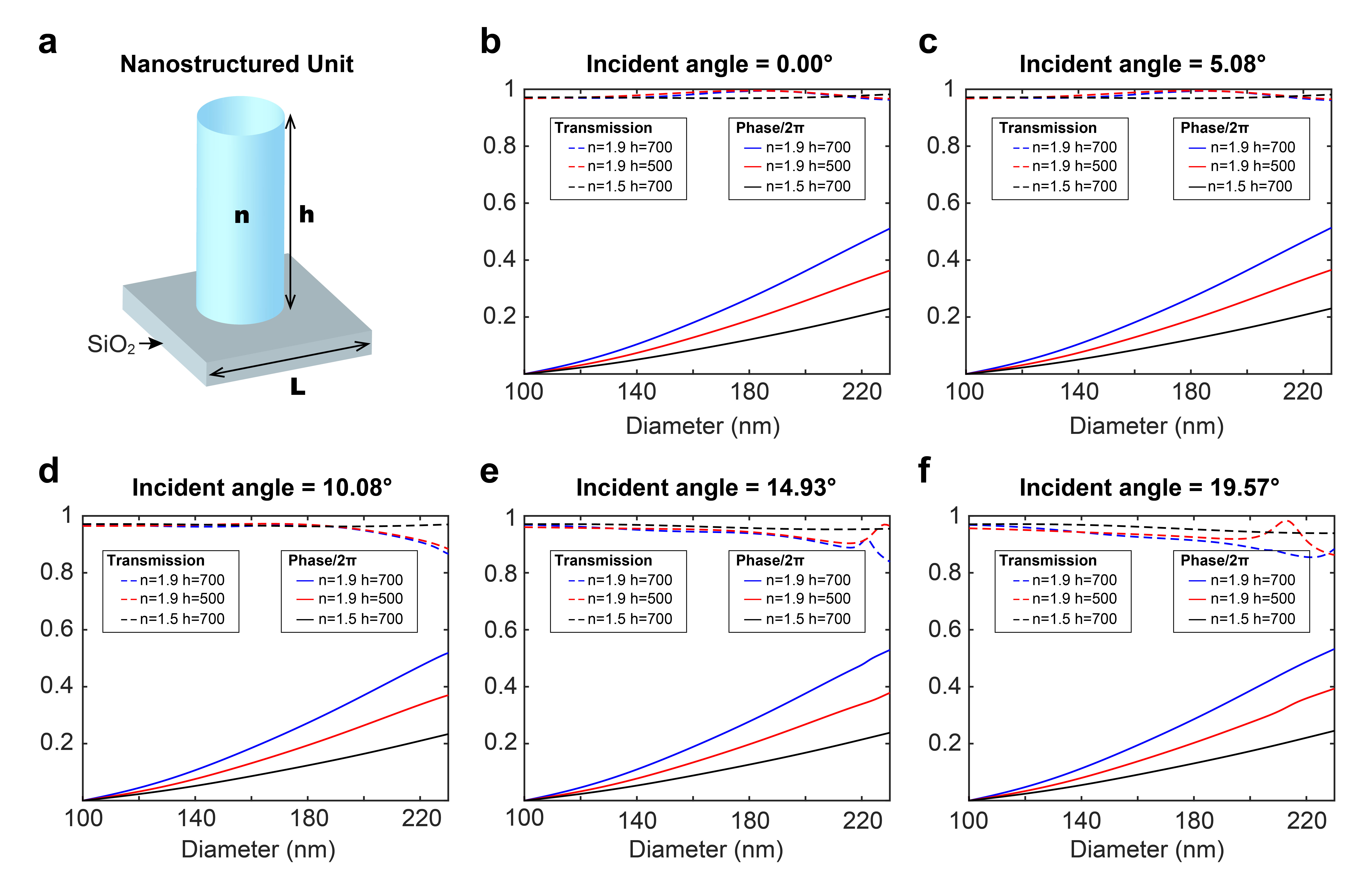


**Fig. S3. Simulation results of the nanopillar grating at 525 nm wavelength. a** Schematic of the nanopillar unit with a lattice period L = 330 nm, where n and h denote the refractive index and height of the nanopillars, respectively. **b-f** Normalized phase and transmittance responses of the nanopillar grating under plane waves incident at different angles. In the plots, dashed lines represent normalized transmittance, solid lines indicate normalized phase, and three colors correspond to three cases: (ⅰ) n=1.9, h=700 nm; (ⅱ) n=1.9, h=500 nm; (ⅲ) n=1.5, h=700 nm.


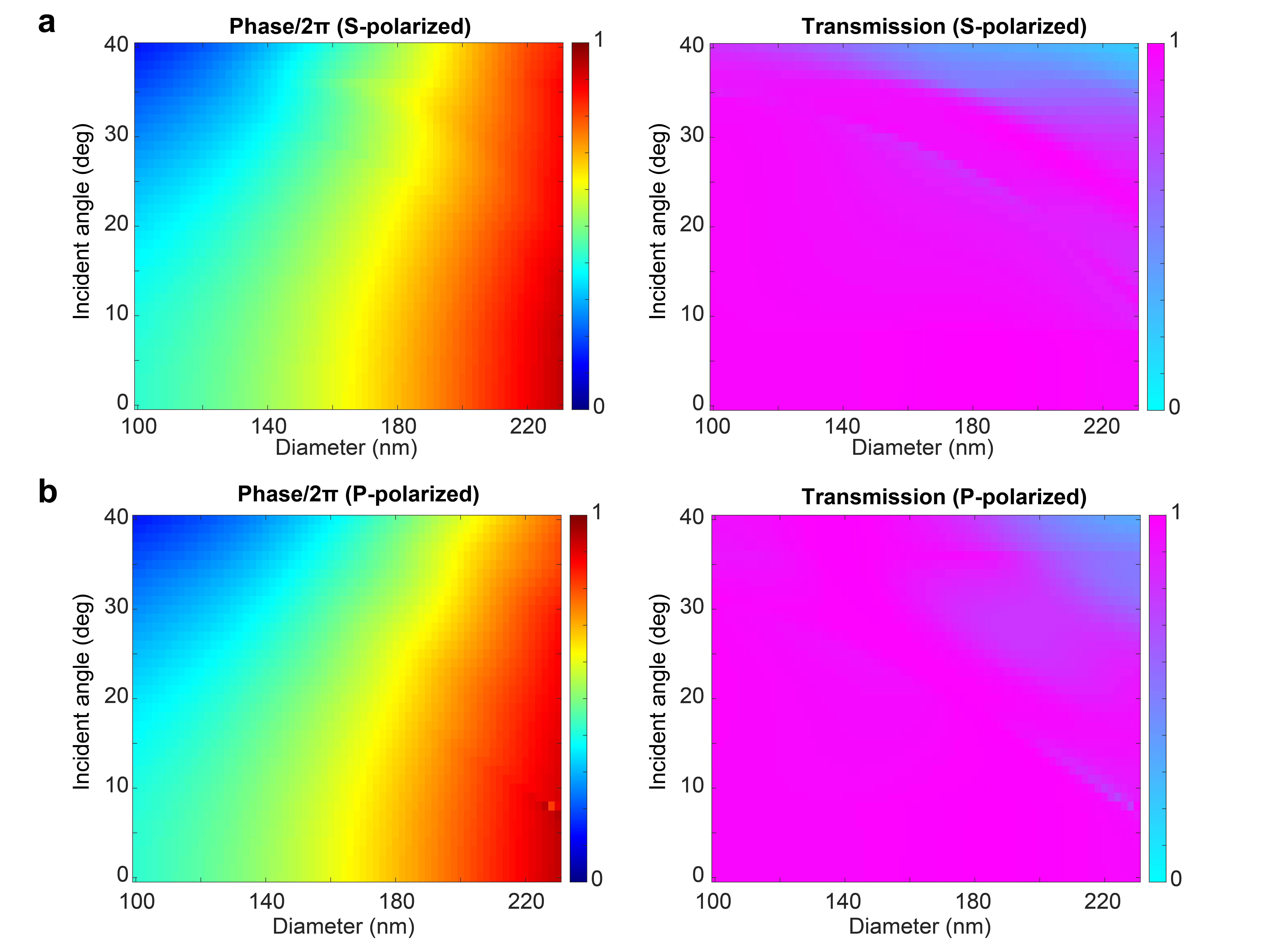


**Fig. S4. Simulated phase and transmission maps of the meta-atoms at 525 nm wavelength.** The phase (left panels) and transmission (right panels) of a cylindrical nanopillar are plotted as a function of diameter and incident angle. The simulations are performed for **a** S-polarized and **b** P-polarized incident light.

##
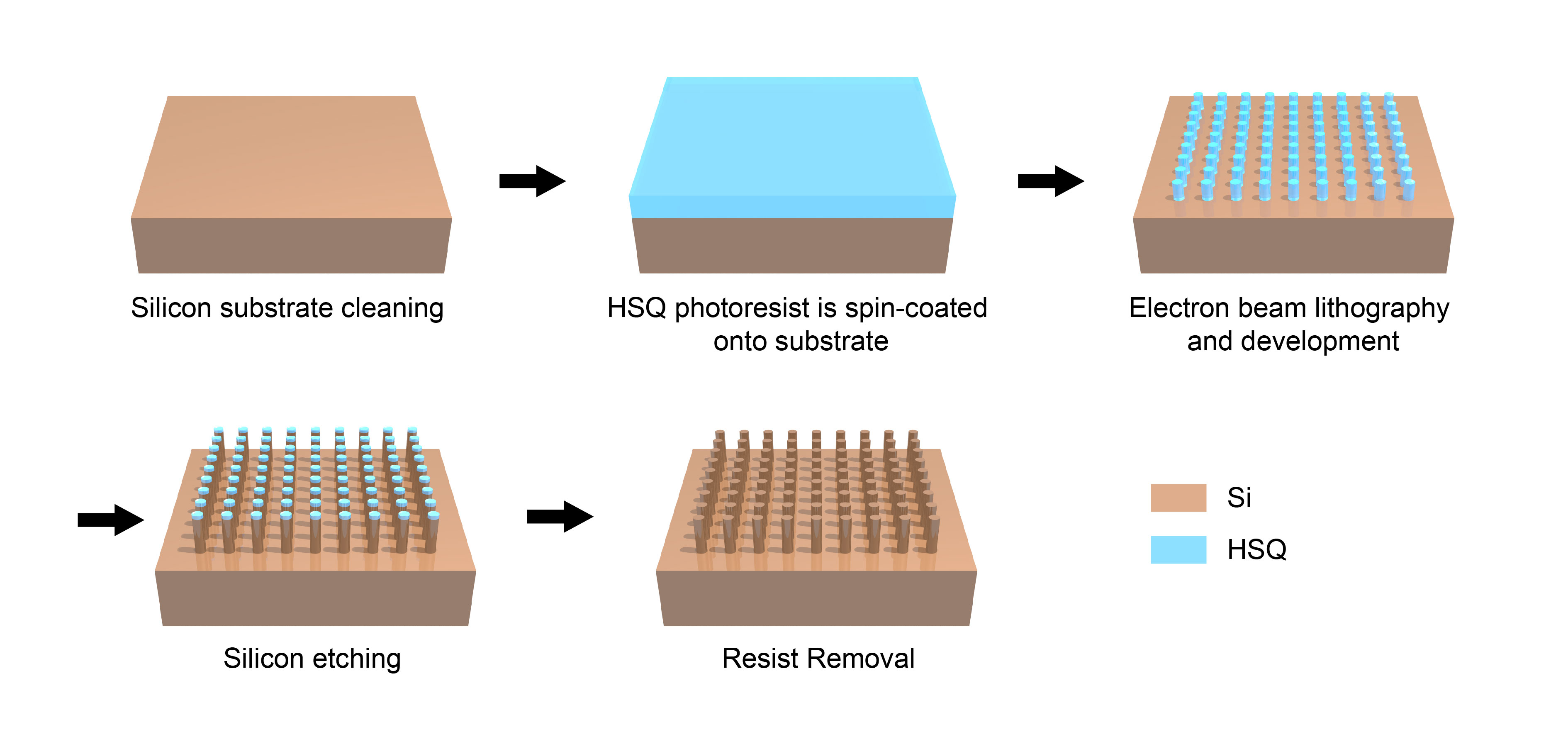


## Fig. S5. Schematic flowchart of the EBL process for fabricating the master mold.

##
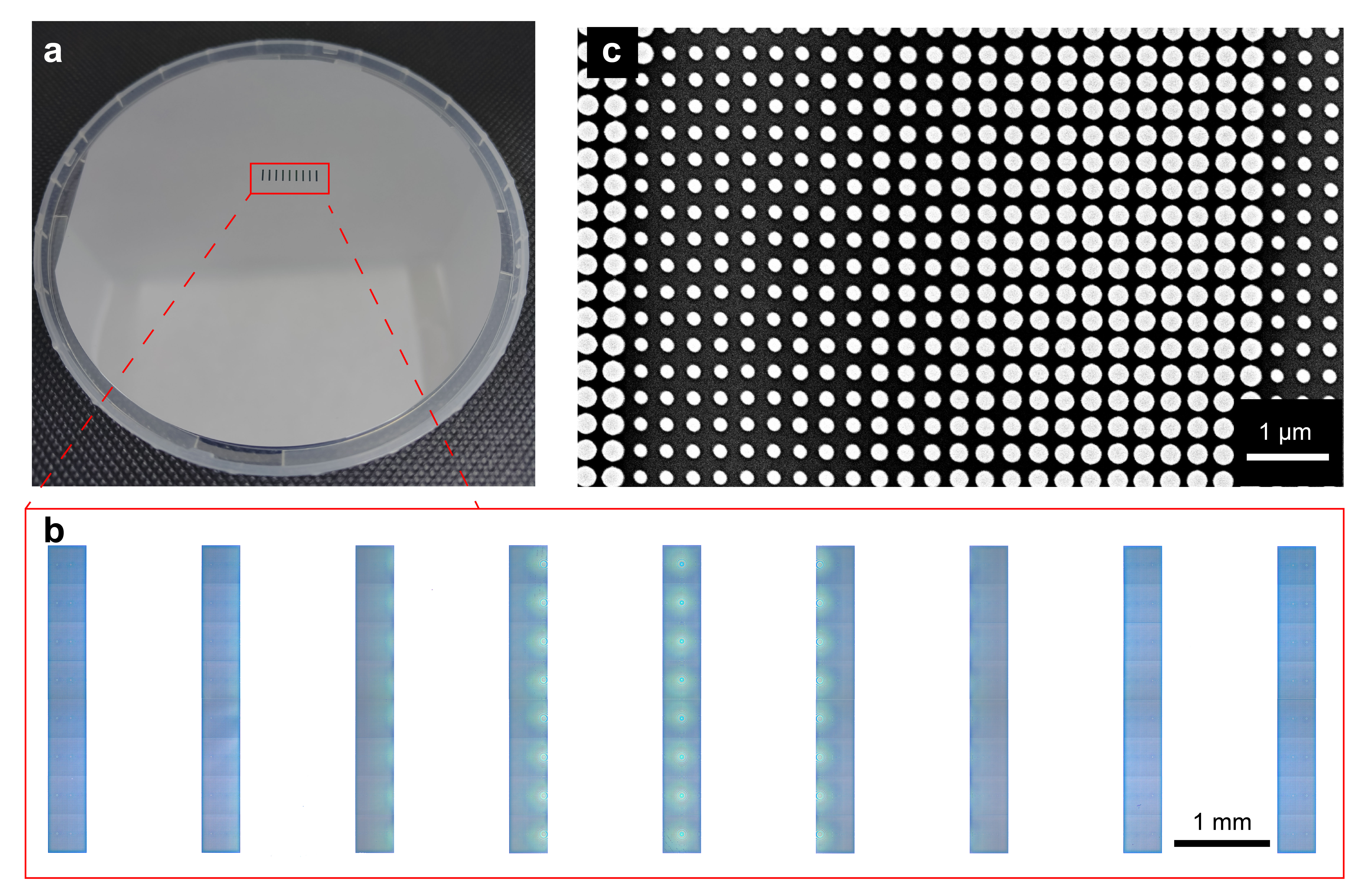


**Fig. S6. The fabricated MeLA nanoimprint silicon master mold.** **a** Photograph of the master mold. **b** Optical microscopy image of the master mold. **c** Top-view SEM image of the master mold.


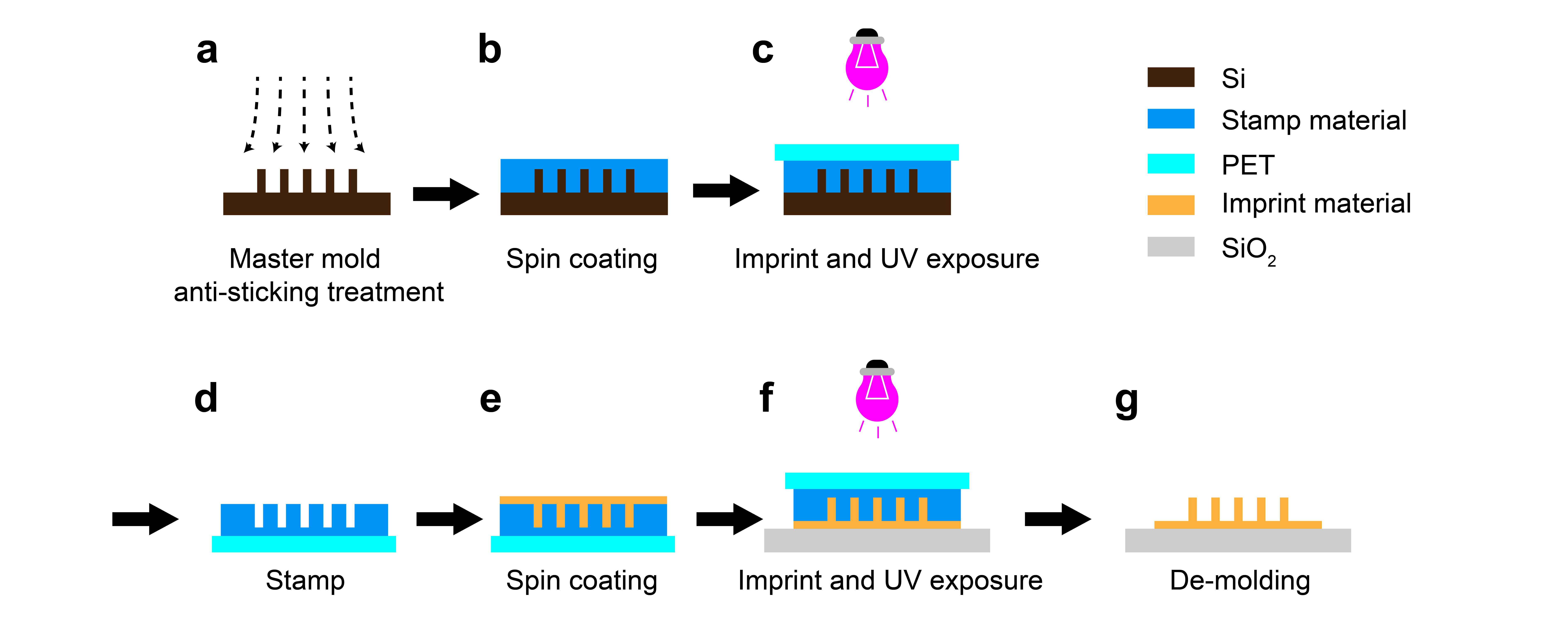


**Fig. S7. Schematic diagram of the MeLA nanoimprint process.**


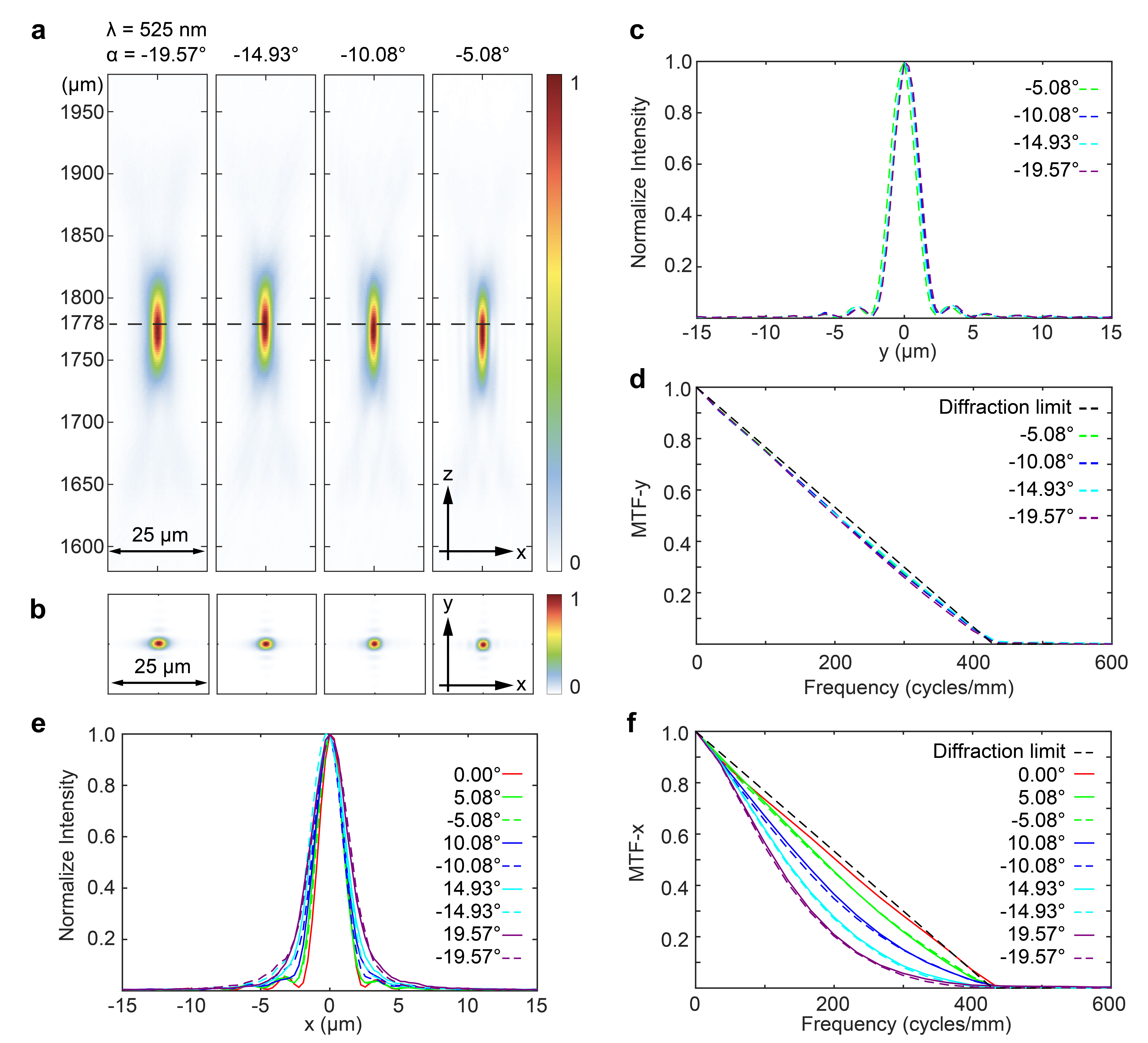


**Fig. S8. Performance characterization of the fabricated MeLA.** **a** Normalized measured intensity distributions in the x-z plane for four different types of MeLA lenslets. **b** Normalized measured intensity distributions at the focal plane corresponding to each lenslet in (**a**). **c** Cross-sectional intensity profiles along the *y*-direction for each focal spot in (**b**). **d** MTFs derived from the curve profiles in (**c**), the black dashed line indicates the diffraction limit. **e** Cross-sectional intensity profiles along the x-direction of the focal spots produced by nine different types of MeLA lenslets. **f** MTFs derived from the profiles in (**e**), the black dashed line marking the diffraction limit.


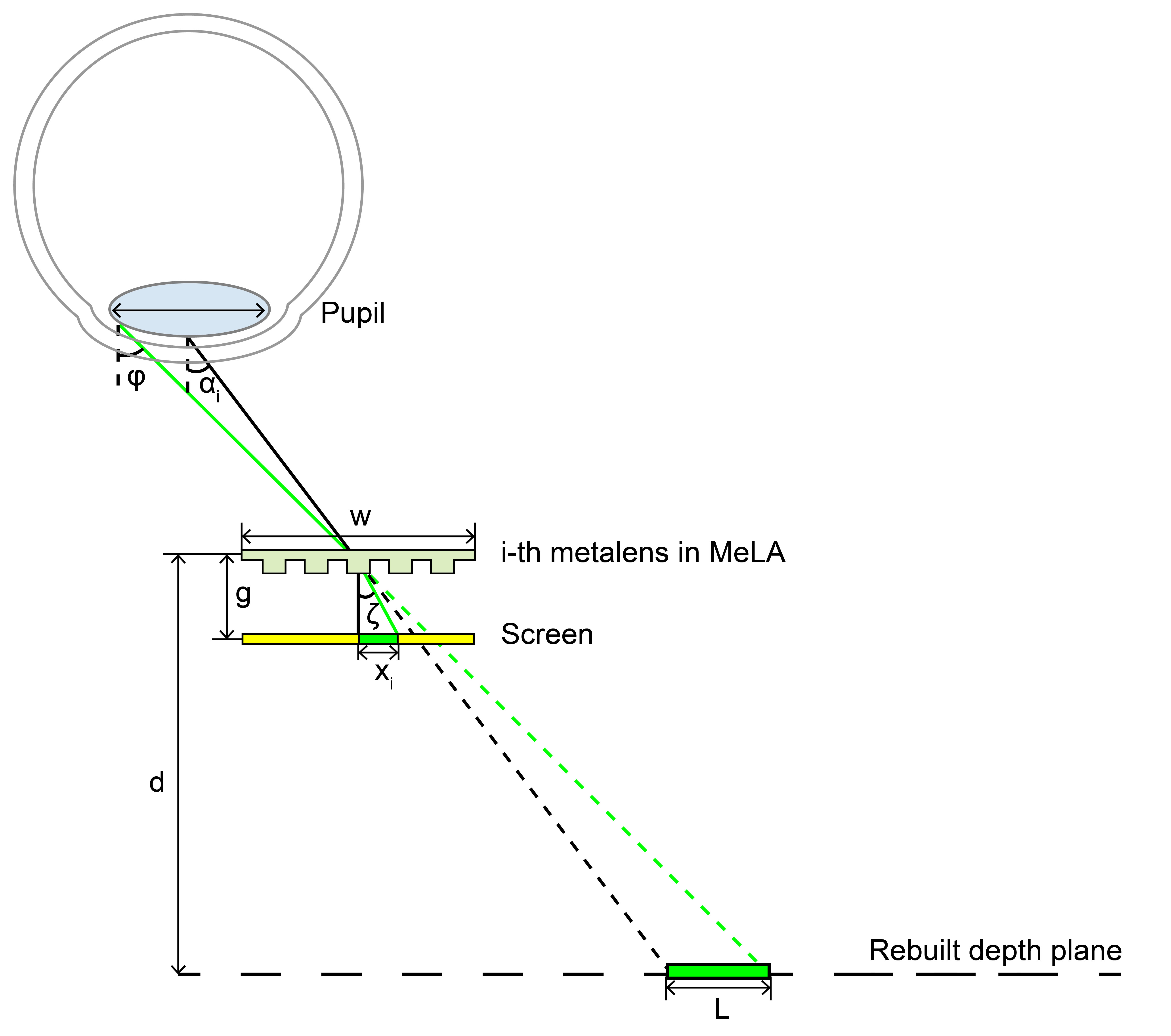


**Fig. S9. Schematic diagram illustrating the nonlinear pixel-voxel mapping relationship in meta-based light-field NED.**


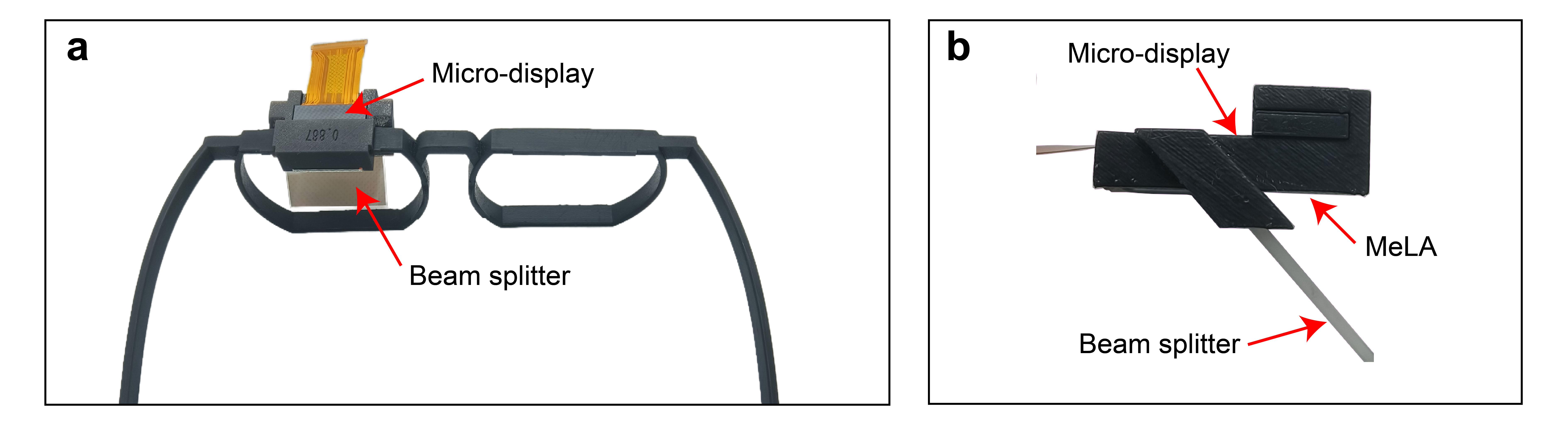


**Fig. S10. The wearable meta-based XR glasses prototype. a** Lightweight frame made with 3D printing. **b** Side view of removable optical module.


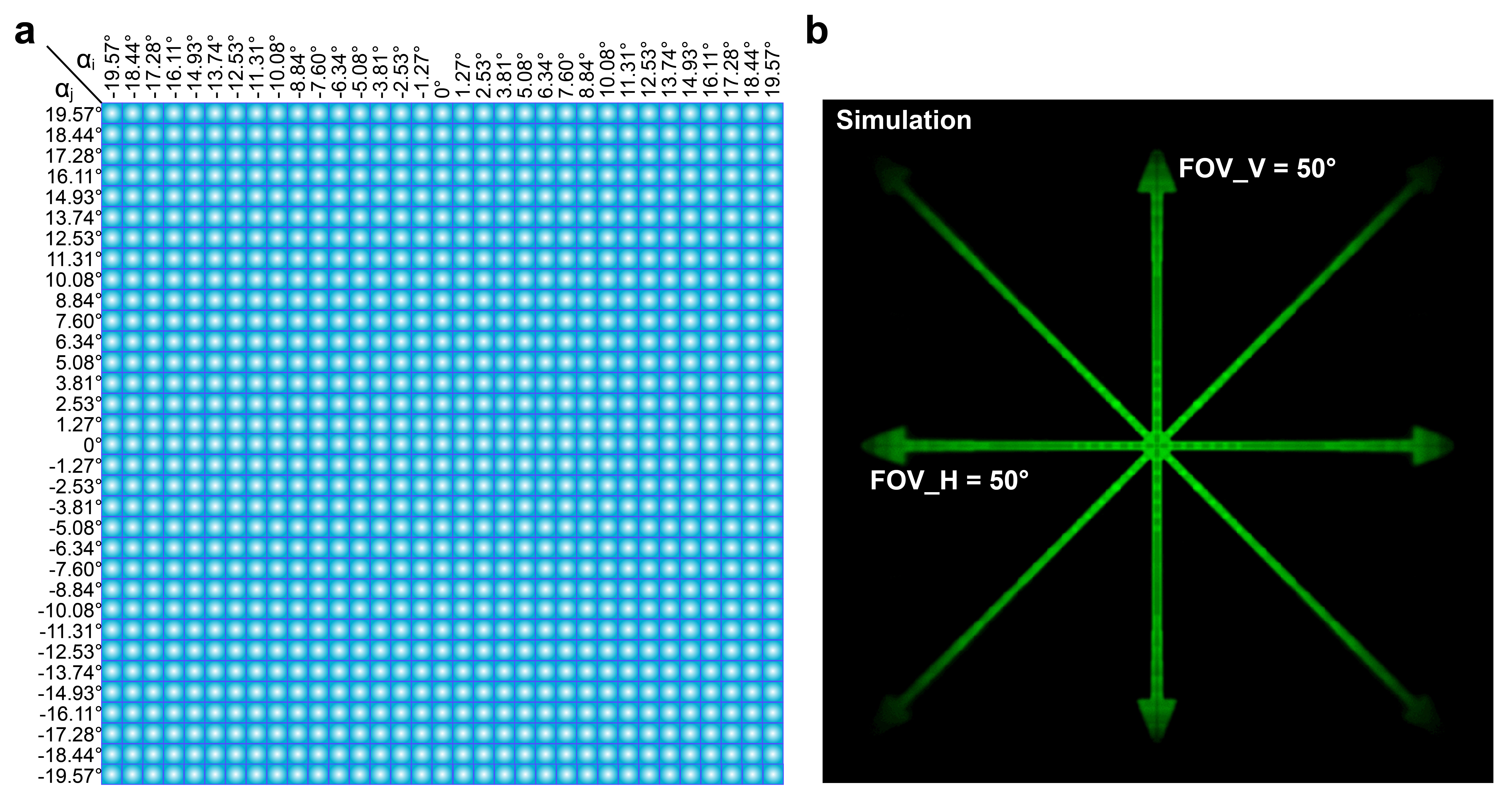


**Fig. S11. Design and simulation of a two-axis FOV expansion. a** An illustration of the distribution of design deflection angles (*αᵢ*, *αⱼ*) across the array. **b** Simulated result of the near-eye display system incorporating the array, demonstrating a symmetrical and continuous 50° × 50° FOV. This confirms the proposed design concept's capability to achieve symmetrical, two-axis FOV expansion.


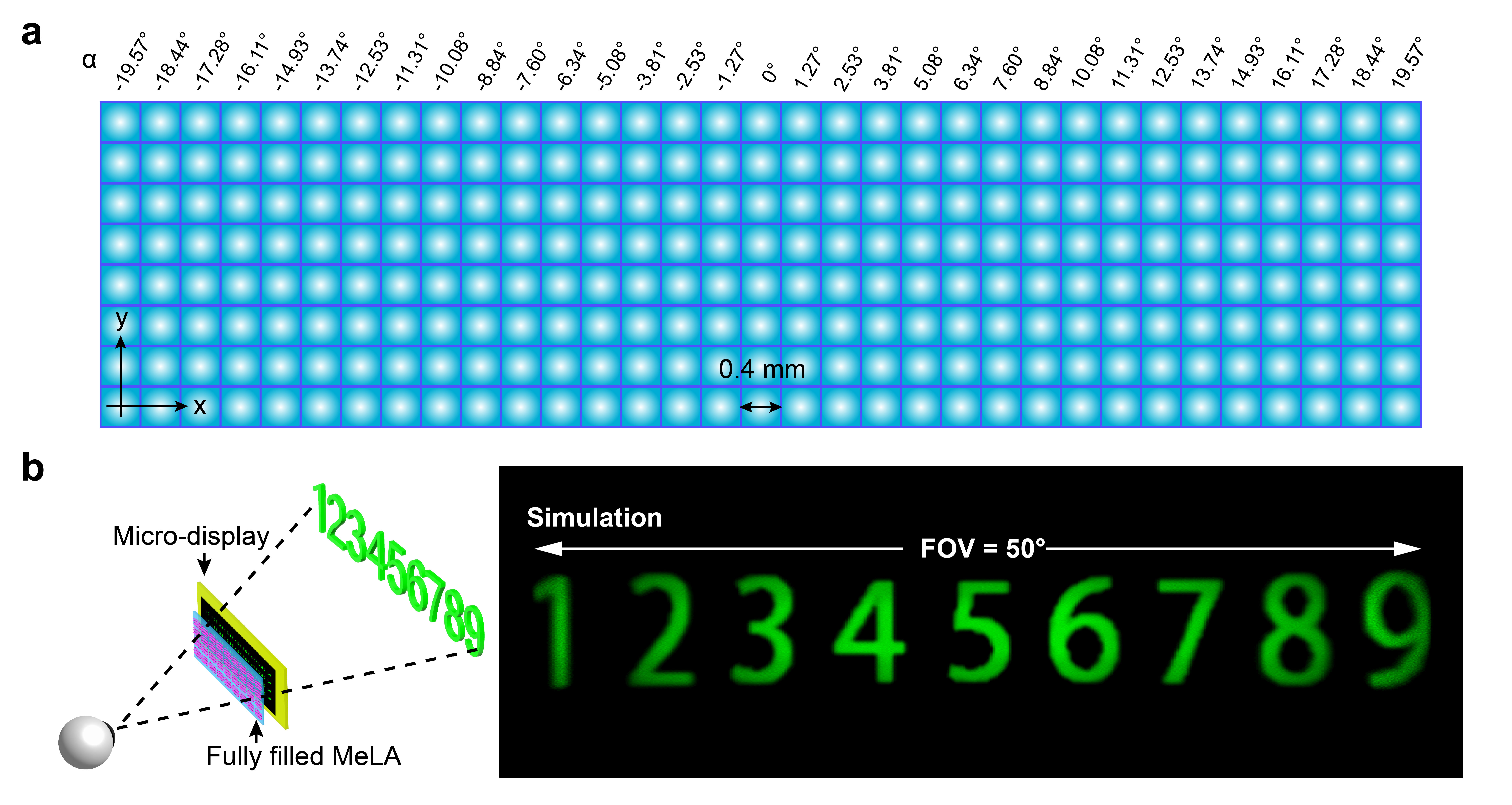


**Fig. S12. Simulation of the FOV for light-field NED based on the fully filled MeLA. a** Schematic of the fully filled MeLA, where *α* denotes the designed deflection angles of each column of metalenses, with a unit width of 400 μm. **b** Schematic of the optical path and the corresponding FOV simulation result. The system achieves an expanded FOV of 50° and successfully displays all digits from 1 to 9.


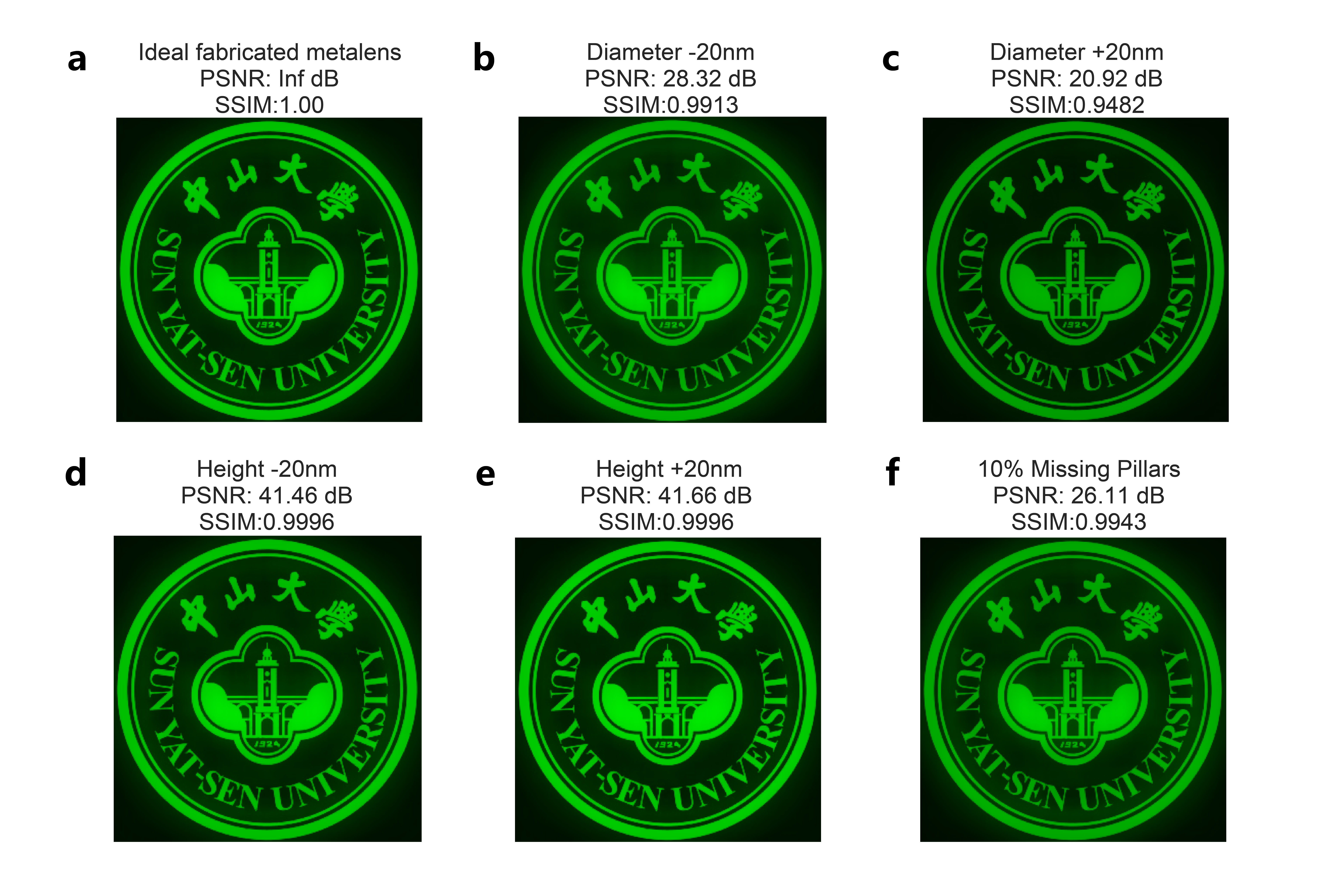


**Fig. S13. Simulation of fabrication tolerance. a** Imaging with an ideal fabricated metalens. **b, c** Imaging with metalenses where all nanopillar diameters are increased by 20 nm and decreased by 20 nm, respectively. **d, e** Imaging with metalenses where all nanopillar heights are increased by 20 nm and decreased by 20 nm, respectively. **f** Imaging with a metalens where 10% of nanopillars are randomly omitted. The PSNR for each case is noted in the corner, showing a manageable degradation in image quality and demonstrating the robustness of the design.


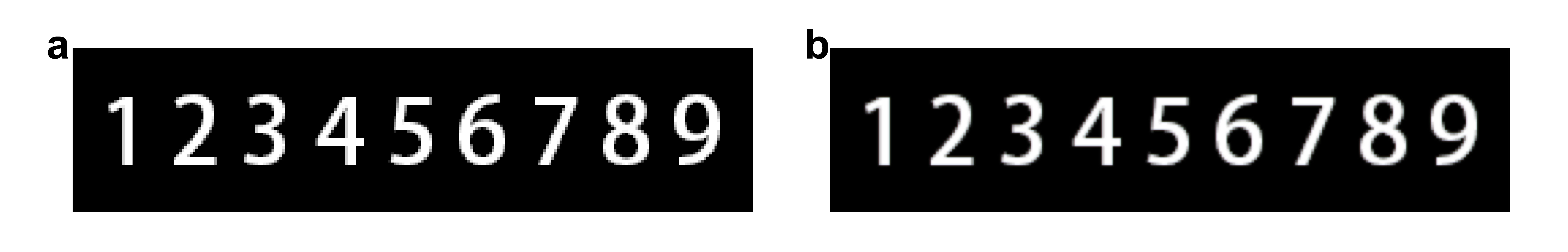


**Fig. S14. Simulated full-color imaging performance of a future achromatic heterogeneous MeLA. a** The original target image. **b** The simulated imaging result under white light illumination. This image was generated by convolving the target image with a set of experimentally measured full-color PSFs obtained from the achromatic metalens work (ref. 40).

**Table S1 Comparison of near-eye display technologies**

|  | Curved microlens array | Metalens eyepiece | Freeform optics with a light field engine | Heterogeneous metalens array |
| --- | --- | --- | --- | --- |
| Reference | IEEE Trans. Vis. Comput. Graph. 26, 1981-1990 (2020). | Sci. Adv. 7, eabe4458 (2021). | Opt. Express 26, 17578-17590 (2018) | This work |
| Display Mode | 2D | 2D | 3D | 3D |
| VAC-Free | No | No | Yes | Yes |
| FOV | 180° | 10° | 30° | 50° |
| Form Factor | Thin | Ultra-thin | Bulky | Ultra-thin |
| Color | RGB | RGB | RGB | RGB (potential) |
| Fabrication | CNC machining/molding | EBL | Ultra-precision single-point diamond turning | Nanoimprint |

**Movie S1**

The virtual character "E" traverses from distant to proximal positions, as illustrated in Fig. 5 of the main text, along the path P_1_→P_2_→P_3_.

**Movie S2**

The virtual character "E" traverses from the right to the left side, as illustrated in Fig. 5 of the main text, along the path P_4_→P_2_→P_5_.
